# Resveratrol alleviates glucolipid metabolism disorders in diabetic nephropathy via the HIF-1α pathway: a network pharmacology and experimental validation study

**DOI:** 10.3389/fphar.2026.1971426

**Published:** 2026-10-30

**Authors:** Siyun Li, Dongxin Lyu, Fuzhen Feng, Runhua Zhou, Jinping Zhang, Yanhui Deng

**Affiliations:** 1 Department of Pharmacy, Jiangmen Central Hospital, Jiangmen, China; 2 Laboratory of Pharmacology & Toxicology, School of Pharmaceutical Sciences, Sun Yat-sen University, Guangzhou, China; 3 Department of Pharmacy, The Sixth Affiliated Hospital, School of Medicine, South China University of Technology, Foshan, China; 4 Department of Pharmacy, The Third Affiliated Hospital of Southern Medical University, Guangzhou, China

**Keywords:** diabetic nephropathy, glucolipid metabolism, HIF-1α, network pharmacology, resveratrol

## Abstract

**Background:**

Diabetic nephropathy (DN) represents a major microvascular complication of diabetes with complex pathogenesis. Resveratrol (RSV), a polyphenol from *Polygonum cuspidatum*, is a potential therapeutic agent for DN. However, the exact mechanisms of RSV modulating glucolipid metabolism in DN remain unclear.

**Methods:**

Potential targets and signaling pathways of RSV against DN were predicted by network pharmacology and molecular docking. Based on the prediction, a DN model was generated in male Wistar rats using a high-fat diet/streptozotocin (HFD/STZ) induction, followed by 12-week RSV (30 mg/kg/d) for *in vivo* validation. *In vitro*, HK-2 cells cultured in palmitic acid (PA) were treated with either RSV or LW6, a hypoxia-inducible factor 1α (HIF-1α) inhibitor. Biochemical parameters, histopathological alterations and lipid deposition were examined. AKT/HIF-1α pathway activation and downstream metabolic gene levels were analyzed via Western blot, immunofluorescence and RT-qPCR.

**Results:**

Bioinformatics findings revealed RSV exerts its anti-DN effects predominantly through the prioritized candidate AKT1 and the highly enriched HIF-1 signaling pathway. *In vivo*, RSV significantly ameliorated hyperglycemia and renal dysfunction and alleviated histopathological injuries and glycogen storage. Mechanistically, RSV suppressed p-AKT and HIF-1α expression, reduced *Glut1* mRNA levels, and elevated *Ldlr* mRNA levels, significantly. *In vitro*, RSV effectively decreased lipid accumulation and inhibited HIF-1α expression, downregulating SREBP1, SREBP2, LDLR, GLUT1 and TGF-β1 and upregulating INSIG2. The effects of LW6 were largely consistent with those of RSV but failed to reduce *HIF-1α* mRNA levels.

**Conclusion:**

The attenuation of glucolipid metabolic disorders in DN by RSV is associated with suppression of the HIF-1α pathway, highlighting the therapeutic potential of RSV for DN.

## 1 Introduction

Genetic and environmental factors are responsible for the development of diabetes mellitus, a chronic metabolic disease characterized by absolute or relative insulin deficiency (Szkudelska and Szkudelski, 2010). Among its various complications, diabetic nephropathy (DN) stands out as a prevalent microvascular complication and main driver of end-stage renal disease (Li et al., 2021). DN pathogenesis is intricate, encompassing glucose metabolic disorders and subsequent inflammation, oxidative stress, lipid metabolic disorders and other comprehensive factors (Forbes et al., 2008). However, there are no satisfactory drugs and treatments at present, which results in high mortality and disability rates in patients with DN.

RSV (3,4′,5-trihydroxystilbene) is a natural polyphenol abundantly found in *Polygonum cuspidatum* (commonly referred to as Hu Zhang in traditional Chinese medicine), with beneficial pharmacological actions, including anti-inflammation, anti-apoptosis and anti-oxidation activities (Jardim et al., 2018). RSV can alleviate insulin resistance, regulate lipid metabolic dysfunction and reduce inflammatory factor expression (Wu et al., 2020). We previously demonstrated that RSV ameliorates hepatic glucolipid metabolism disorders in diabetic rats through suppressing the 3-phosphoinositide-dependent protein kinase 1 (PDK1)/AKT phosphorylation and HIF-1α expression (Li et al., 2023). Given that the kidney is also a critical organ for glucolipid homeostasis, we hypothesized that RSV might exert similar effects in DN. Other *in vivo* studies have reported that RSV could ameliorate metabolic disorders and renal impairment by modulating glucolipid metabolism through multiple signal pathways (Kim et al., 2013; Um et al., 2010; Zhao and Fan, 2020). Because of the characteristics of RSV with multiple targets and pathways, its molecular mechanism for regulating glucolipid metabolism in DN warrants further investigation.

Network pharmacology, which integrates systems biology and polypharmacology, provides a holistic framework for elucidating drug mechanisms through the construction of drug-target-disease interaction networks. It has also transformed the traditional concept of “one target, one drug, one disease” into a novel version (Hopkins, 2008). Additionally, molecular docking serves as a computational approach commonly employed to predict binding modes and affinities between small-molecule ligands and related disease targets, which can be utilized to efficiently screen key targets that bind to active ingredients (Huang and Zou, 2010).

Therefore, we conducted network pharmacology analysis and molecular docking to reveal the underlying mechanisms of RSV for modulating glucolipid metabolism in DN, followed by experimental validation *in vivo* and *in vitro*. The overall workflow is summarized in Figure 1.

**FIGURE 1:**
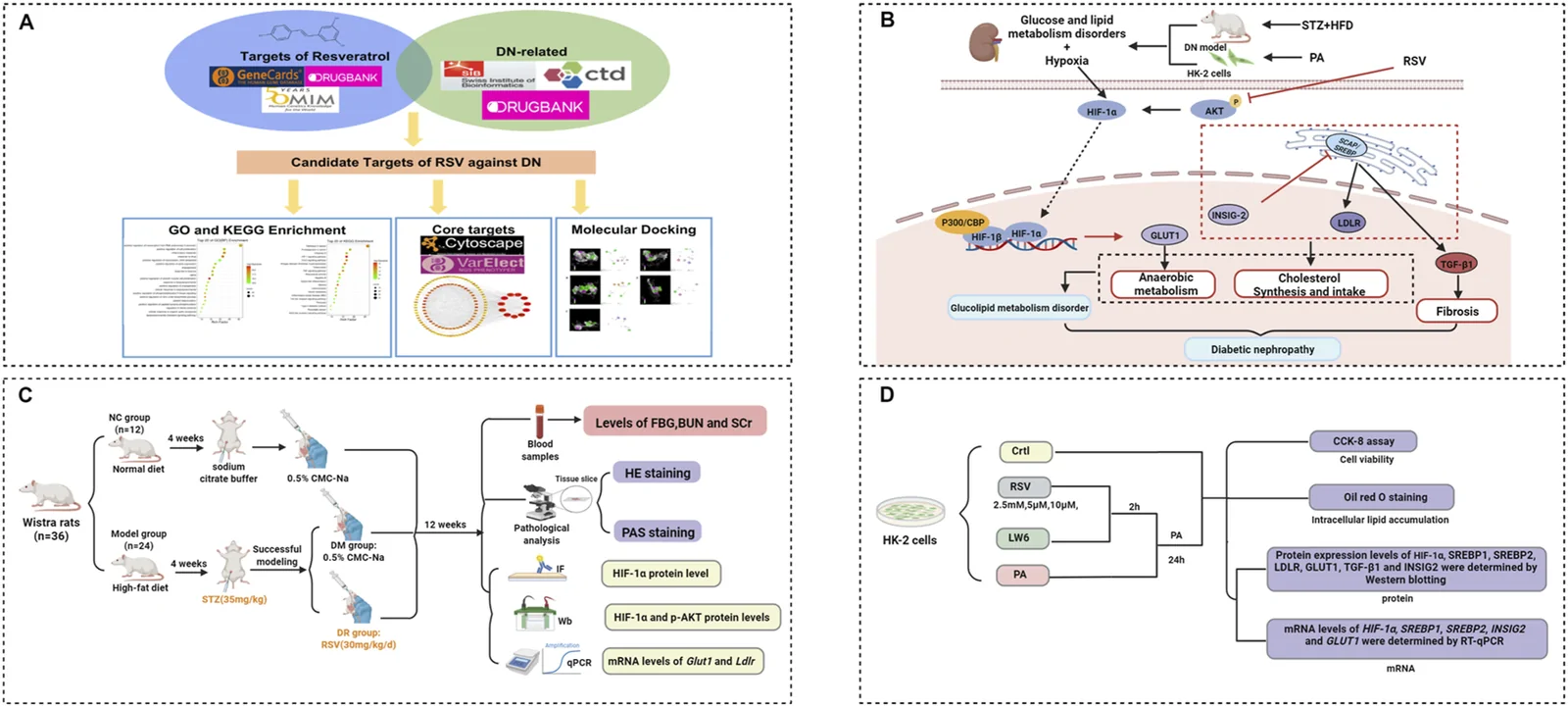
Experimental design and mechanistic diagram depicting the protective role of RSV in ameliorating glucolipid metabolism disorders in DN through the HIF-1α pathway. **(A)** Bioinformatics approaches predicted AKT1 as a prioritized candidate and the HIF-1 signaling pathway as the top enriched pathway. **(B)** The mechanistic hypothesis proposed that RSV attenuates glucolipid metabolism disorders in DN via the HIF-1α pathway. **(C)** *In vivo* experiments validated the mechanism in DN rats. **(D)** *In vitro* experiments validated the mechanism in PA-treated HK-2 cells.

## 2 Materials and methods

### 2.1 Network pharmacology

Canonical SMILES of RSV were sourced from PubChem. We searched three databases to obtain targets of resveratrol: Swiss TargetPrediction, Drugbank and Comparative Toxicogenomics Databases. DN-related genes were acquired from Online Mendelian Inheritance in Man (OMIM), Genecards and Drugbank databases by using the search terms “diabetic kidney disease” and “diabetic nephropathy”. The common targets of RSV-related genes and DN-related genes were obtained by Venn diagram analysis (Venny 2.1) and retrieved from UniProt (species: *Homo sapiens*). The outcomes were considered candidate therapeutic targets of RSV against DN.

Common targets were imported into STRING 11.0 then visualized by the Cytoscape 3.7.0 software for the purpose of predicting protein-protein interaction (PPI, confidence >0.700). The top 10 ranked targets regarding degree and betweenness centrality were selected. To analyze gene-phenotype correlation, the overlapping targets were uploaded to VarElect. The top 10 direct and indirect genes were selected to evaluate and identify the prioritized candidates. Finally, the intersection of the top 10 ranked targets from both topological and gene-phenotype analyses was considered as the prioritized candidates of RSV against DN.

The intersecting targets were imported into the DAVID online tool and analyzed by Gene Ontology (GO) and Kyoto Encyclopedia of Genes and Genomes (KEGG) enrichment analysis (*P* < 0.01). Then a network model of target-pathway interaction was constructed based on these results using Cytoscape 3.7.0. Finally, the pathways highly enriched and related to glucolipid metabolism regulation in DN were considered as potential signal pathways.

### 2.2 Molecular docking

Molecular docking was aimed at estimating molecular interactions between prioritized candidates and RSV. The three-dimensional structures of prioritized candidates and RSV were respectively acquired from Protein Data Bank database (PDB) and ZINC. Moreover, their structure files were imported into Discovery Studio 2016 and AutoDock Tools 1.5.6 (Trott and Olson, 2010), then the docking was conducted running Autodock Vina with CMD command character. The affinity was calculated to evaluate docking between ligands/resveratrol and targets, and the docking poses were visualized by Discovery Studio 2016.

### 2.3 Experimental validation

#### 2.3.1 Animal experimental design

All animal experiments were performed under the ethical approval of the Southern Medical University Animal Ethics Committee (Guangzhou, China, No. L2020066) in accordance with the China Animal Welfare Legislation. Five-week-old male Wistar rats (110–140 g) from the Laboratory Animal Centre of Southern Medical University were kept under specific pathogen-free (SPF) conditions (24 °C ± 2 °C, 45% ± 5% humidity, 12-h light/dark cycle). Following acclimation, animals received either a standard chow diet (normal control [NC] group, n = 12) containing 13% fat (No. 1016706476803973120, Beijing KAXL FEED) or an HFD containing 45% fat (Cat. No.1236463, Beijing HFK) for 28 days. After a 12-h fasting period, intraperitoneal injection of STZ (35 mg/kg) freshly dissolved in 0.1 M cold citrate buffer (pH 4.5) was used to generate diabetes (Gheibi et al., 2017), while NC rats received a constant volume of sodium citrate buffer. Within 72 h after STZ (Sigma-Aldrich, St. Louis, MO, United States) injection, rats with fasting blood glucose (FBG) concentrations higher than 16.7 mmol/L were considered diabetic. FBG was determined from tail-tip blood samples utilizing the glucometer (Accu-Chek Performa, Roche, GER).

After successful modeling, we randomly assigned the diabetic rats to either diabetic model group (DM, n = 12) or RSV-treated group (DR, n = 12). These 2 groups remained on the HFD, whereas the NC group received a normal chow diet for 12 weeks continuously. Resveratrol (purity >99%, HPLC) from Shanghai Winherb Medical Technology Co. Ltd. (Shanghai, China) was dissolved in 0.5% carboxymethyl cellulose sodium (CMC-Na) at 3 mg/mL and administered to DR rats via oral gavage at 30 mg/kg/day, 6 days per week. The other two groups were given an equivalent volume of vehicle. The RSV dosage was converted from the effective dosage in the clinical trials (250 mg/d) (Bhatt et al., 2012; Den and Tsiani, 2019; Nair and Jacob, 2016) and further verified by previous rodent studies (Wang et al., 2017; Wang et al., 2016). The equation for extrapolation of dose between humans and rats: Animal equivalent dose (mg/kg) = Human dose (mg/kg) × Km ratio (Km ratio of rats is 6.2) (Nair and Jacob, 2016). After the 12-week treatment period, overnight-fasted animals were weighed and then sacrificed under anesthesia. Blood samples were collected, and serum was separated and stored at −20 °C. Kidneys were then harvested and weighed. Kidney tissues were prepared for histopathological and immunofluorescence analysis, while renal cortex sections were stored at −80 °C for Western blotting and RT-qPCR analysis.

#### 2.3.2 Biochemical parameters

BUN (Blood urea nitrogen) and SCr (Serum creatinine) concentrations were assayed using an automatic biochemical analyzer in the Department of Laboratory Medicine of the Third Affiliated Hospital of Southern Medical University.

#### 2.3.3 Kidney staining analysis

To assess kidney morphology and renal glycogen distribution, after fixation in 4% paraformaldehyde, the kidneys were paraffin-embedded, sectioned (4 μm), and subjected to HE and PAS staining (Solarbio) for histological analysis. The images were captured utilizing a light microscope (Model BX43, Olympus).

#### 2.3.4 Immunofluorescence

Renal sections were incubated with the anti-HIF-1α primary antibody (1:200, Cell Signaling Technology), followed by the Alexa Fluor 488 goat anti-rabbit IgG antibody (1:500, Invitrogen). Nuclei were stained with blue-fluorescent DAPI (Sigma-Aldrich, United States). The images were acquired at ×400 magnification using a fluorescence microscope and FV10-ASW software.

#### 2.3.5 Cell culture and treatments

Human renal tubular epithelial (HK-2) cells were kindly provided by Dr. Heqing Huang (School of Pharmaceutical Sciences, Sun Yat-sen University). STR profiling was subsequently performed and the full report is attached as a Supplementary Material. We maintained HK-2 cells in low-glucose Dulbecco’s modified Eagle’s medium (DMEM, Gibco, C11885500BT) supplemented with 10% fetal bovine serum (FBS) in 5% CO_2_ at 37 °C. To mimic an optimal high-fat (HF) condition, cells were exposed to varying PA (Sigma-Aldrich, United States) concentrations (0, 50, 100, 150, 200 and 250 μM) for 2 h, followed by cell viability assessment. The effects of RSV on HF-stressed HK-2 cells were assessed across an RSV concentration range of 0–80 μM (0, 1.25, 2.5, 5, 10, 20, 40 and 80 μM). After starvation for 12 h in low-glucose DMEM supplemented with 0.5% FBS, cells in the PA + RSV groups and the PA + LW6 group were pre-treated for 2 h with either RSV or 20 μM LW6 (an HIF-1α inhibitor, Selleck, Houston, TX, United States), respectively. Subsequently, HK-2 cells in the PA + RSV groups and the PA + LW6 group were exposed to the selected concentration of 150 μM PA for 24 h.

#### 2.3.6 Cell viability assay

Cell viability was evaluated by a Cell Counting Kit-8 (CCK-8) detection Kit (M0856, MIKX, Shenzhen, China). HK-2 cells seeded into 96-well plates were treated with graded concentrations of PA or RSV for 24 h. Subsequently, 10 μL CCK-8 solution was added to each well and incubated for an additional 2 h under standard culture conditions (37 °C, 5% CO_2_). To calculate the cell viability relative to the control group, the absorbance value was then measured at 450 nm.

#### 2.3.7 Cellular Oil Red O staining

Lipid accumulation in HK-2 cells was evaluated by a commercial Oil Red O staining kit (C0157S, Beyotime Biotechnology) following the standardized protocol. Images were subsequently acquired with a cell imaging system.

#### 2.3.8 Quantitative real-time PCR analysis

Total RNA was extracted from kidneys and HK-2 cells with TRIzol reagent (Takara), and 1 μg of total RNA per sample was used for subsequent analysis. Reverse transcription was conducted using the PrimeScript™ RT Master Mix (Perfect Real Time) Kit (Takara) for kidneys and the SweScript RT II First Strand cDNA Synthesis Kit (Servicebio) for cells. RT-qPCR was quantified utilizing the ChamQ SYBR qPCR Master Mix (High ROX Premixed, Vazyme) with a 20 μL reaction volume for kidneys and 2×Polarsignal SYBR Green qPCR mix (MKG800-10, MIKX) for cells, subsequently carried out using the StepOne Plus Real-Time System.

Relative gene expression was normalized to β-actin and calculated with the 2^−ΔΔCT^ method.

#### 2.3.9 Western blotting

Western blotting was conducted as previously described (Li et al., 2023). Primary antibodies against p-AKT (1:1,000) and GLUT1 (1:1,000) were both purchased from Cell Signaling Technology. Antibodies against INSIG-2 (1:1,000), SREBP1 (1:1,000), SREBP2 (1:1,000) and TGF-β1 (1:5,000) were from Proteintech. Antibodies against HIF-1α (1:500), LDLR (1:1,000) and β-actin (1:2000) were obtained from Immunoway, Abcam and Servicebio, respectively.

#### 2.3.10 Statistical analysis

Data were expressed as mean ± standard error of the mean (SEM) and analyzed utilizing GraphPad Prism 9.5. All other data analyses were performed using one-way ANOVA followed by the LSD t-test. The values of *P* < 0.05 were considered statistically significant. Exact *P* values and effect sizes are provided in Supplementary Table S1.

## 3 Results

### 3.1 Network pharmacology and molecular docking

#### 3.1.1 Candidate targets of RSV against DN and prioritized candidates screening

As shown in Figure 2A and Tab.1, candidate targets of RSV in treatment of DN were derived from 130 common genes (two non-human genes were deleted in Tab.1). Then the PPI network (Figure 2B) was constructed using the STRING 11.0 database (confidence >0.7). After excluding one common target (SLC22A6) that had no interactions, the final network consisted of 127 nodes and 1,245 edges (average degree 19.6).

**FIGURE 2:**
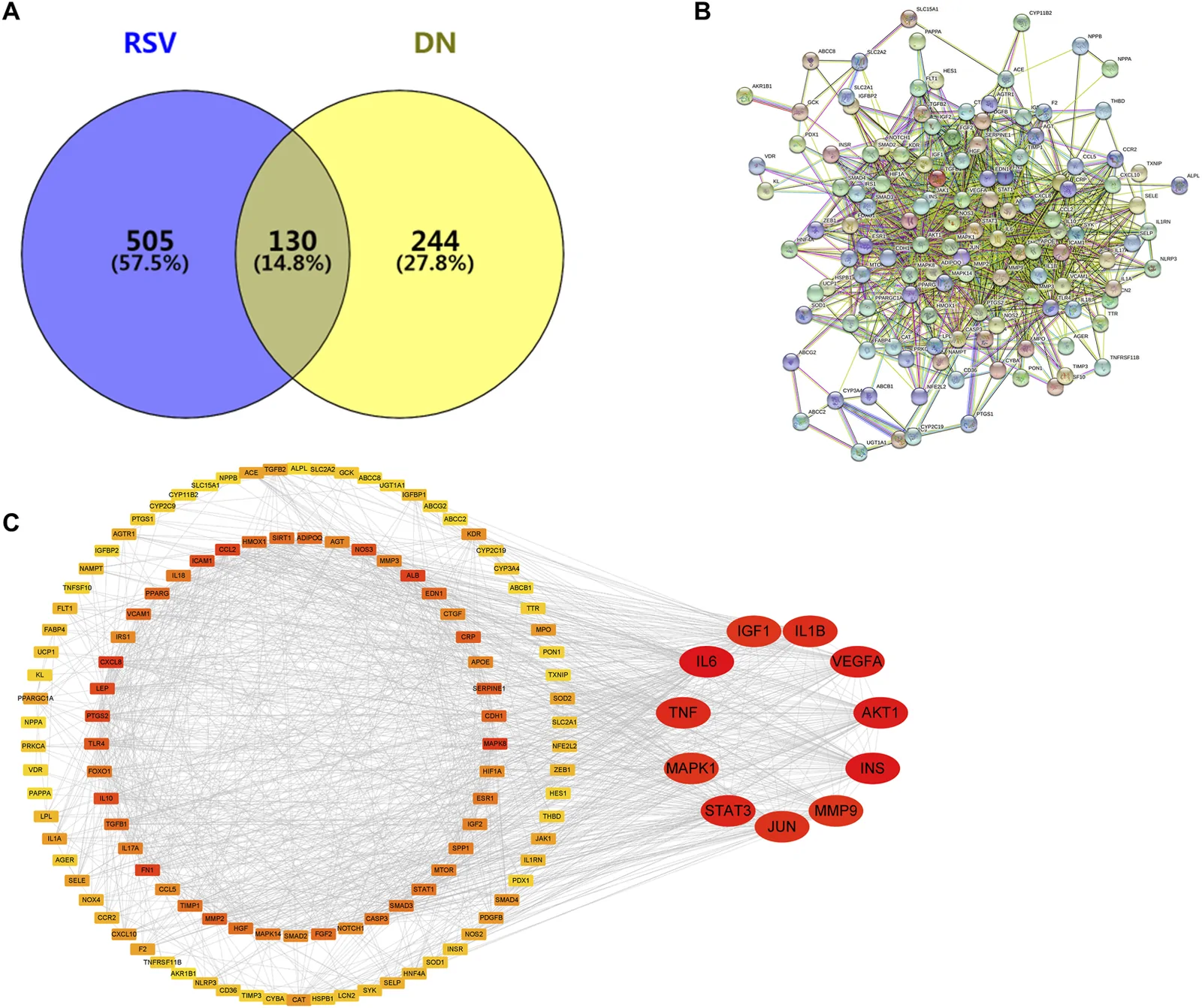
Candidate targets of RSV against DN and prioritized candidates screening. **(A)** Intersection of resveratrol and DN targets. **(B)** PPI network of resveratrol targets in DN. **(C)** Topological analysis of the targets of resveratrol against DN. Nodes represent targets and edges represent interactions. Red nodes represent the top 10 targets, with darker shades reflecting higher degree.

**TABLE 1:** Degree and betweenness centrality of top 10 ranked targets (with ties at rank 9).

Rank	Target	Degree	Betweenness centrality
1	IL6	72	1,187.7
2	INS	67	2,359.7
3	AKT1	64	1,087.3
4	VEGFA	60	629.2
5	STAT3	55	544.9
6	TNF	51	455.0
7	IL1B	49	397.8
8	JUN	48	356.2
9	IGF1	47	624.4
9	MAPK1	47	484.7
9	MMP9	47	396.1

As depicted in Figure 2C and Tab.1, targets were ranked by degree using the Network Analyzer in Cytoscape 3.7.0. The top 10 ranks (including ties) comprised 11 targets: IL6, INS, AKT1, VEGFA, STAT3, TNF, IL1B, JUN, MMP9, IGF1 and MAPK1. Among these, three targets (IGF1, MAPK1, and MMP9) were tied at rank 9 with the same degree value of 47, thus resulting in a total of 11 targets. In addition, the results of VarElect analysis indicated that 57 targets were directly and 70 targets were indirectly related to the DN phenotype. Both top 10 directly and indirectly relevant genes regarding average disease-causing likelihood were shown in Tab.2. Therefore, the prioritized candidates are AKT1, IL1B, INS, JUN and STAT3.

**TABLE 2:** Top 10 directly or indirectly DN-associated genes ranked by average disease-causing likelihood (gene-phenotype relevance).

Symbol	Relationship	Average disease causing likelihood (%)
GCK	Direct	92.5
AKT1	Direct	90.8
SLC2A1	Direct	88.7
MAPK8	Direct	87.0
STAT1	Direct	84.7
ALB	Direct	82.3
IL1B	Direct	81.8
MTOR	Direct	80.4
INSR	Direct	75.6
INS	Direct	74.5
SMAD2	Indirect	91.1
PRKCA	Indirect	89.4
HES1	Indirect	84.8
TGFB2	Indirect	82.8
SMAD4	Indirect	82.3
JAK1	Indirect	81.8
JUN	Indirect	79.9
HSPB1	Indirect	79.7
IL1RN	Indirect	79.1
STAT3	Indirect	78.9

#### 3.1.2 GO and KEGG enrichment

To clarify the underlying mechanism of common targets, they were submitted to the DAVID database for GO and KEGG pathway analysis. As shown in Figure 3A, GO enrichment analysis indicated shared genes might act on cellular components, such as extracellular space, extracellular region and platelet alpha granule lumen, then exert molecular functions, such as cytokine activity, growth factor activity and identical protein binding. Additionally, in biological processes analysis (Figure 3C), target genes were mainly enriched in positive regulation of smooth muscle cell proliferation, response to drug and positive regulation of nitric oxide biosynthetic process, which were tightly linked to cell proliferation and inflammation.

**FIGURE 3:**
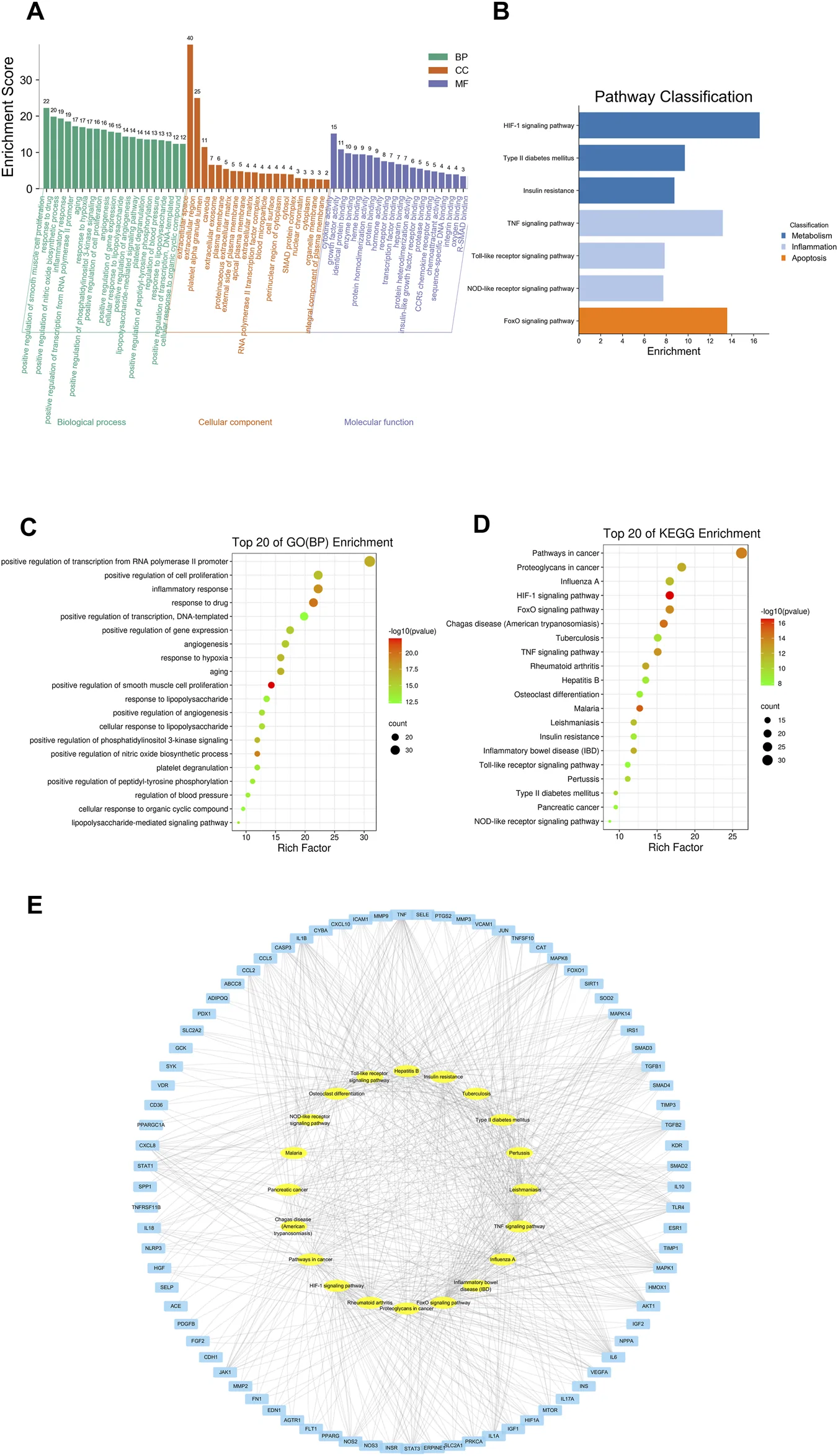
GO and KEGG enrichment analysis. **(A)** Top 20 information of GO enrichment analysis. **(B)** Pathway classification of resveratrol for regulating glucose and lipid metabolism in DN. **(C)** Top 20 bubble chart of the biological process. Color scale: P-value threshold; dot size: gene count per term. **(D)** Top 20 bubble chart of KEGG pathway enrichment analysis. Color scale: P-value threshold; dot size: gene count per term. **(E)** Target-signal pathway network diagram of resveratrol against DN. Blue nodes and yellow nodes represent targets and pathways, respectively.

The target-signaling pathway network was rendered with Cytoscape (Figure 3E). In addition, seven signaling pathways were related to glucose and lipid metabolism in DN with high enrichment scores (Figure 3D). Among them, the enrichment score and significance of the HIF-1 signaling pathway were the highest. Furthermore, classification of the above signaling pathways indicated that most of them were closely associated with inflammation and metabolism, which could be applied to predict the mechanism of RSV for regulating glucolipid metabolism in DN (Figure 3B).

#### 3.1.3 Molecular docking

Molecular docking served as a computational approach for evaluating binding activity between resveratrol and prioritized candidates and elucidating their binding modes (Figure 4). A lower binding affinity value indicates stronger binding activity between the compound and the targets. Consequently, an affinity value below −5.0 and −7.0 kcal/mol was defined as good and strong binding activity, respectively (Zhang et al., 2021). As shown in Tab.3, the affinity between resveratrol and each prioritized candidate was less than −5.0. The affinity between RSV and AKT1 was the lowest. Moreover, the binding activity between RSV and INS, IL1B and JUN was better than that between the original ligand and the above three targets. Therefore, RSV probably interacted strongly with the identified prioritized candidates.

**FIGURE 4:**
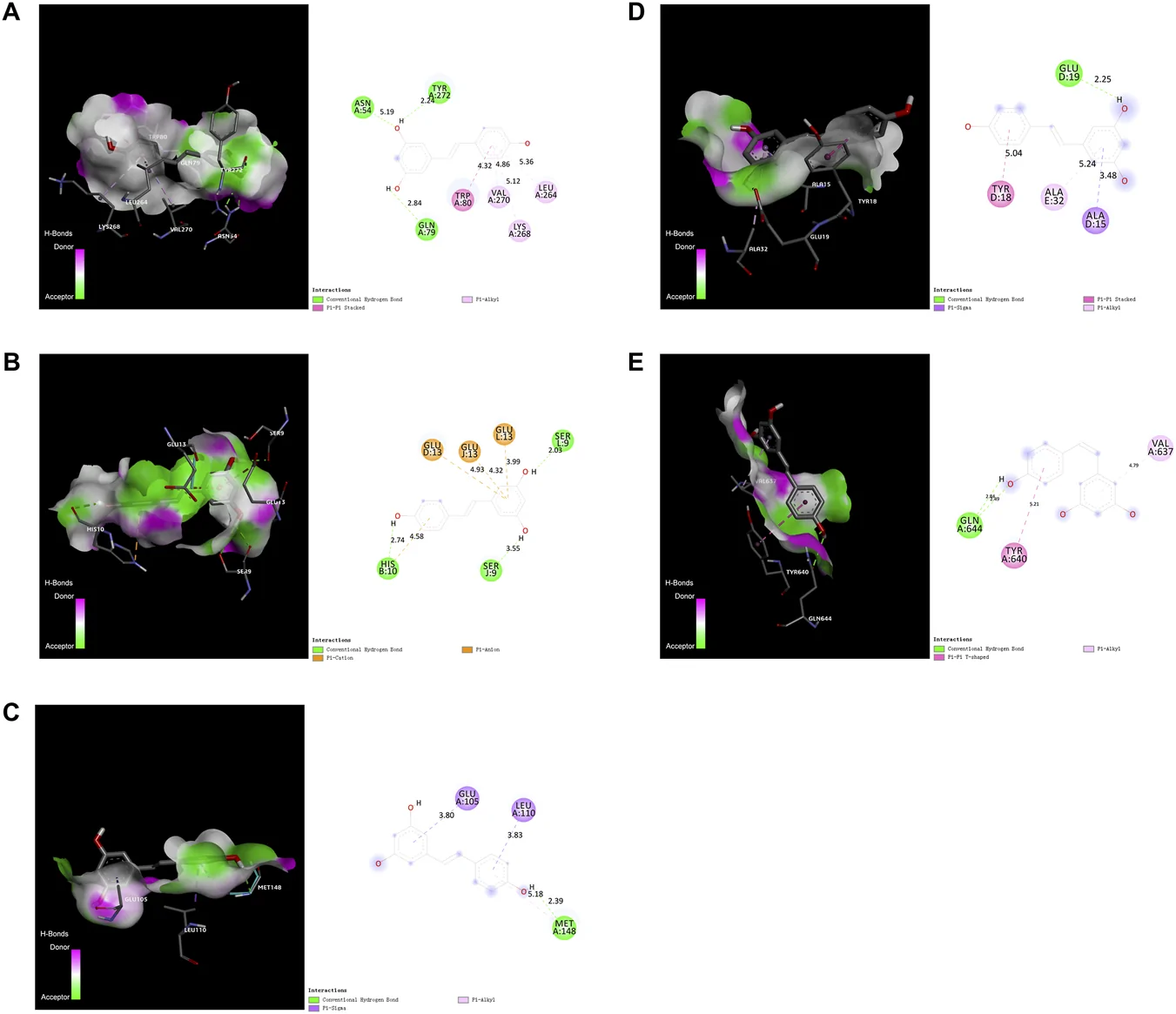
Molecular docking model. **(A)** AKT1-RSV; **(B)** INS-RSV; **(C)** IL1B-RSV; **(D)** JUN-RSV; **(E)** STAT3-RSV. Pictures with a black background and a white background show three-dimensional and two-dimensional models, respectively. Different colors in a two-dimensional model represent the different interactions between resveratrol and targets.

**TABLE 3:** Results of molecular docking.

Target	Affinity (kcal/mol)	
	Ligand	Resveratrol
AKT1	−7.6	−12.5
INS	−7.1	−5.1
IL1B	−6.1	−4.4
JUN	−5.6	−3.8
STAT3	−5.0	−6.4

Although HIF-1α was not among the prioritized candidates identified by topological or VarElect analysis, AKT1, the top-affinity docking hit, is a well-established upstream regulator of HIF-1α protein translation through the PI3K/AKT pathway (Zeng et al., 2022). We therefore focused on HIF-1α as the downstream effector of AKT signaling in subsequent experiments.

### 3.2 RSV protected against renal dysfunction and renal lipid deposition in DN rats

#### 3.2.1 RSV improved general characteristics and ameliorated renal function in DN rats

To assess the protective role of RSV in overall status and renal function in DN rats, body weight, FBG, BUN and SCr were measured. After modeling for 12 weeks, compared with NC rats, kidney index (KI), FBG, BUN and SCr were increased significantly, while body weight was decreased significantly in DN rats (Figures 5A–E). The FBG was higher than 16.7 mmol/L in DN rats. Fortunately, FBG, BUN and SCr were decreased significantly by RSV treatment in the DR group. However, body weight and KI remained significantly unchanged in RSV-treated DN rats (*P* > 0.05). Overall, successful establishment of the DN rat model was achieved, and RSV decreased FBG with ameliorating renal function in DN rats.

**FIGURE 5:**
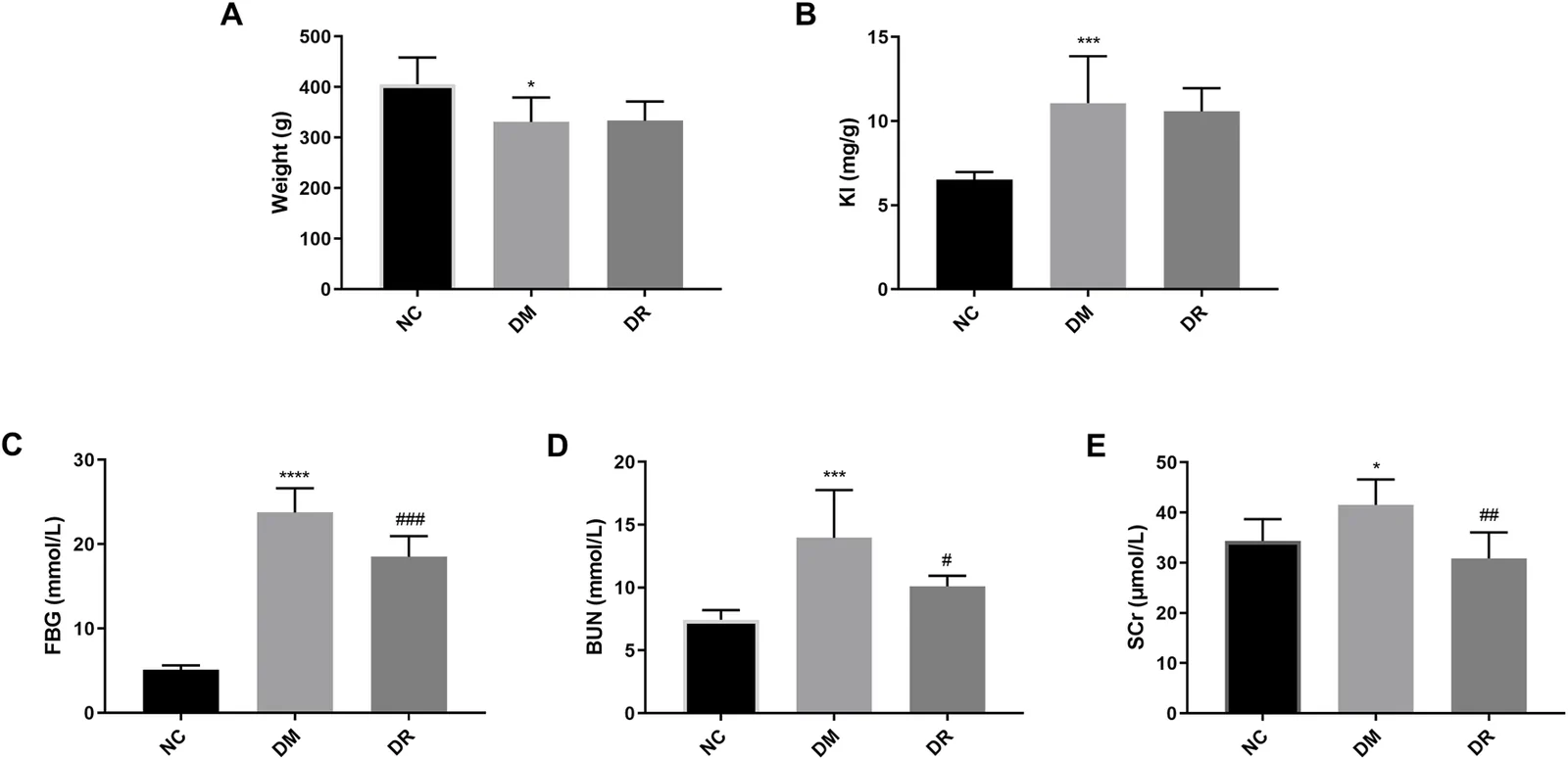
General characteristics and renal function indices of rats (n = 6/group). **(A)** Body weight. **(B)** Kidney index. **(C–E)** Levels of FBG, BUN and SCr, respectively. ^*^ *P* < 0.05, ^**^ *P* < 0.01, ^***^ *P* < 0.001, ^****^ *P* < 0.0001 vs NC group; ^#^ *P* < 0.05, ^##^ *P* < 0.01, ^###^ *P* < 0.001, ^####^ *P* < 0.0001 vs DM group.

#### 3.2.2 RSV reduced the renal lipid deposition of DN rats

The kidney is essential for glucose metabolism and reabsorption, so its pathological injuries can affect blood glucose levels. The HE staining analysis indicated that the kidneys from DN rats showed increased glomerular volume, glomerular mesangial cell proliferation and mesangial matrix expansion, as well as renal tubular basement membrane thickening and tubular atrophy, accompanied by renal interstitial focal inflammatory cell infiltration (Figure 6A). After 12 weeks of RSV intervention, these pathological alterations were effectively ameliorated.

**FIGURE 6:**
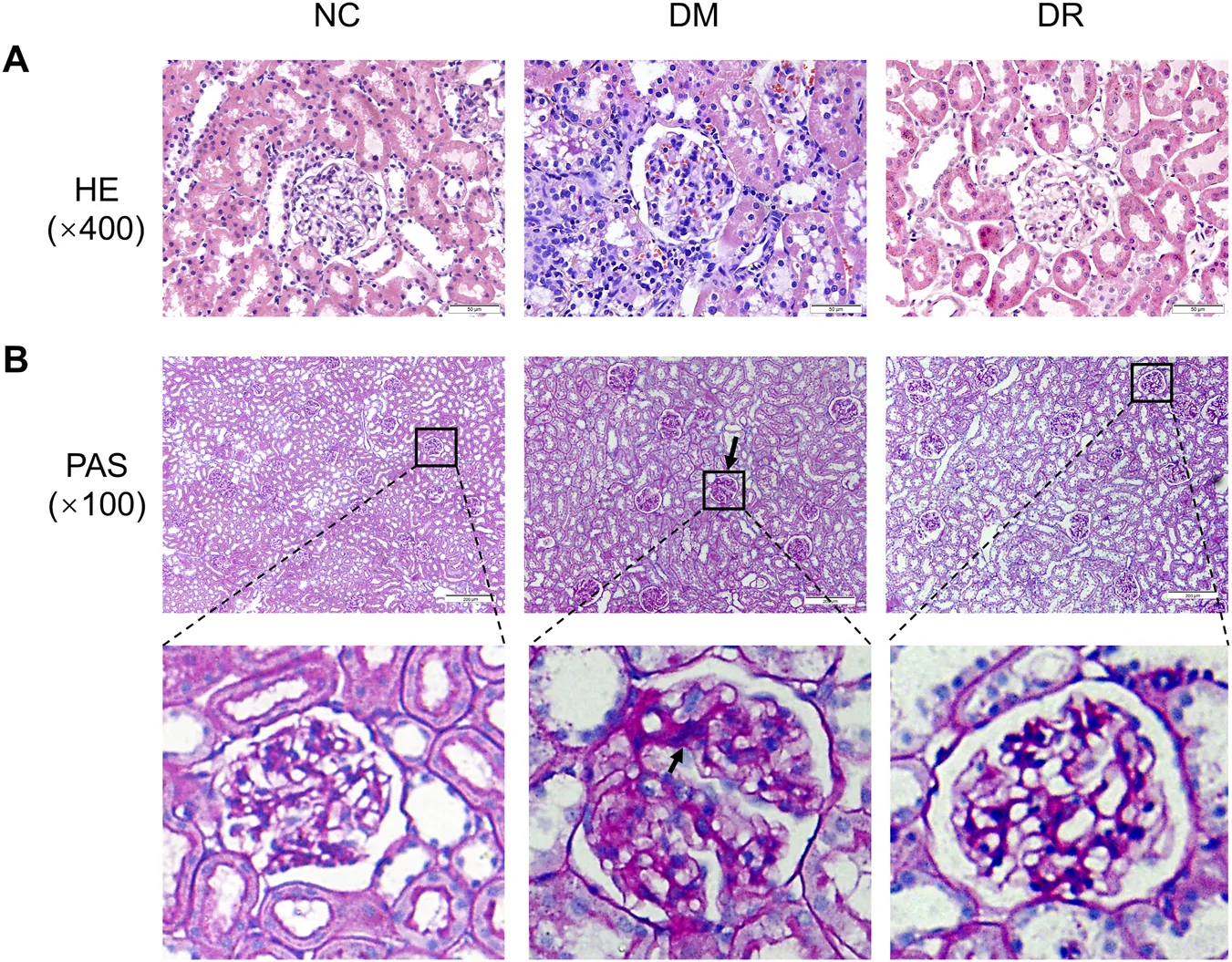
RSV protected against renal injuries in DN rats. **(A)** HE staining. Magnification, ×400; Scale bar = 50 μm. **(B)** PAS staining. Black arrows indicate PAS-positive granules. Magnification, ×100; Scale bar = 200 μm; n = 6.

The PAS staining showed significantly increased accumulation of polysaccharides and glycogen in DN rats (black arrows) compared with control, suggesting that renal glucose metabolism was abnormal. However, PAS-positive granules were significantly reduced in DR group (Figure 6B), indicating that RSV improved renal glucose metabolism and reduced the accumulation of glycogen.

### 3.3 RSV reduced p-AKT and HIF-1α protein levels and regulated downstream metabolic genes in DN rats

Based on the KEGG enrichment results, the relationship between the HIF-1 signal pathway and DN was explored in this study. Protein levels were measured by immunofluorescence and Western blotting. Fluorescence intensity and protein levels of HIF-1α were markedly enhanced in the DM group versus the NC group. However, both of them were significantly downregulated after RSV treatment (Figures 7A–C), showing that glucolipid metabolism disorders induced HIF-1α upregulation and RSV attenuated renal injury by inhibiting HIF-1α expression in DN rats. AKT acts upstream of the HIF-1 pathway associated with DN progression, and molecular docking results identified that AKT1 displayed the highest binding affinity with RSV. Therefore, the protein level of p-AKT was measured to further determine whether RSV’s inhibitory effect on HIF-1α expression was attributable to reduced AKT phosphorylation. As depicted in Figure 7D, although p-AKT expression was significantly higher in the diabetic rats than in the normal rats, the increment was significantly reversed by RSV treatment. These results indicated that RSV reduced p-AKT levels, possibly reflecting decreased AKT phosphorylation, which may in turn lower HIF-1α expression. However, total AKT was not assessed in this study.

**FIGURE 7:**
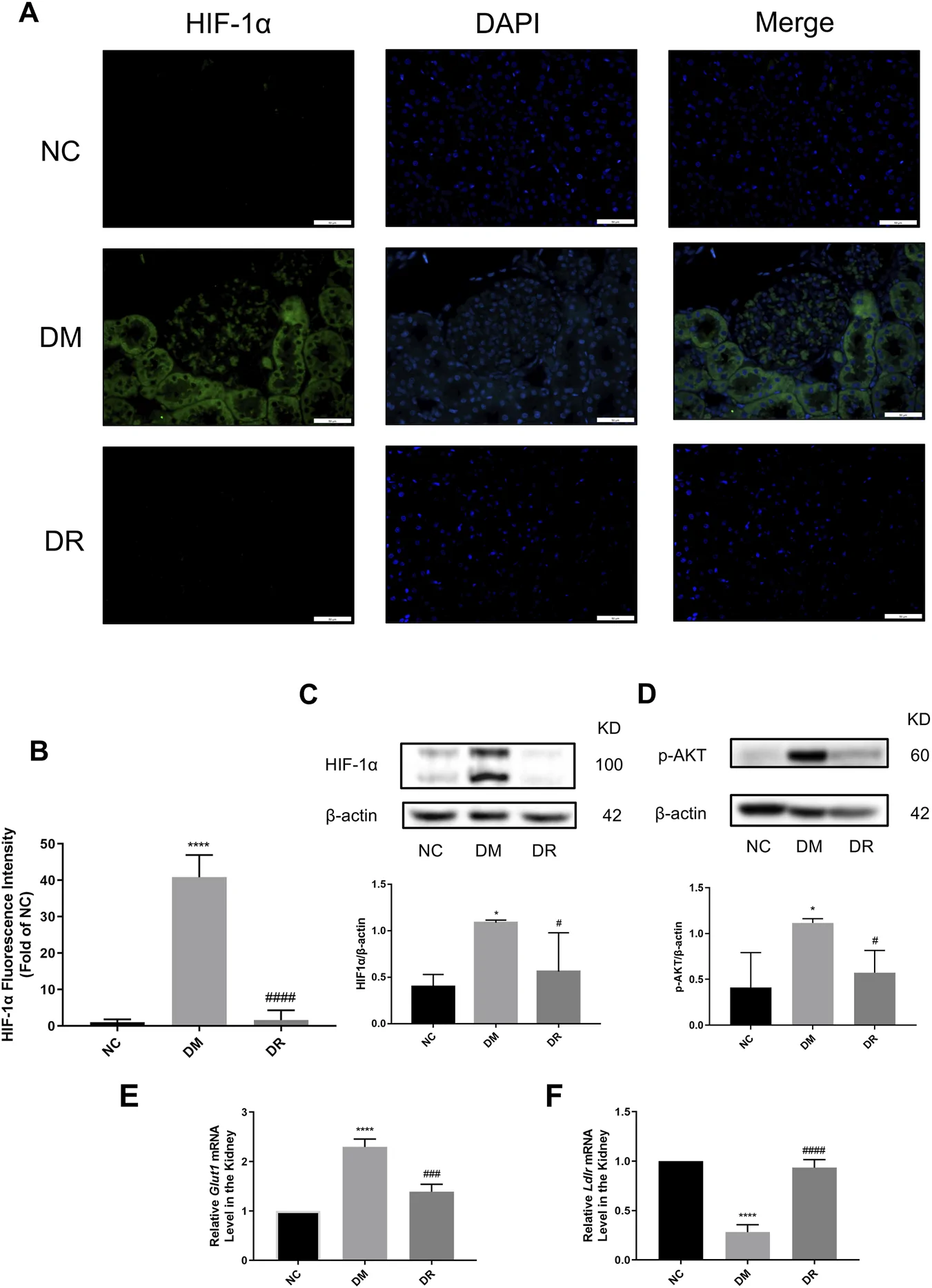
RSV inhibited p-AKT/HIF-1α protein expression and the *Glut1* mRNA level but enhanced the *Ldlr* mRNA level in DN rats. **(A)** HIF-1α protein level was measured by immunofluorescence. Magnification, ×400; Scale bar = 50 μm. **(B)** Quantification of HIF-1α protein fluorescence intensity. **(C–D)** HIF-1α and p-AKT protein levels were measured by Western blotting, respectively. **(E–F)** *Glut1* and *Ldlr* mRNA levels were measured by RT-qPCR, respectively. ^*^ *P* < 0.05, ^**^ *P* < 0.01, ^***^ *P* < 0.001, ^****^ *P* < 0.0001 vs NC group; ^#^ *P* < 0.05, ^##^ *P* < 0.01, ^###^ *P* < 0.001, ^####^ *P* < 0.0001 vs DM group; n = 3.

To further elucidate whether RSV regulates glucolipid metabolism by affecting HIF-1α downstream genes, we measured the mRNA levels of *Glut1* and *Ldlr* in the kidney. In DM rats, the mRNA level of *Glut1* was markedly elevated, while the mRNA level of *Ldlr* was markedly reduced compared with the NC rats (Figures 7E,F). After RSV treatment, the *Glut1* level was reduced and the *Ldlr* level was enhanced, significantly. These findings indicated that RSV could regulate genes involved in glucolipid metabolism, with a potential association with HIF-1α.

### 3.4 RSV attenuated PA-induced lipid accumulation in HK-2 cells via the HIF-1α pathway

#### 3.4.1 RSV alleviated PA-induced HK-2 cells damage

To further clarify the *in vitro* actions of RSV modulating glucolipid metabolism in DN, HK-2 cells were treated with a series of PA concentrations (50–250 μM), simulating the HF environment of DN. As shown in Figure 8A, higher PA levels significantly decreased cell viability. Following stimulation with 150 μM PA, a marked decline in cell viability was detected compared with the untreated cells. Additionally, intracellular lipid droplets stained with Oil Red O indicated that lipid storage was obviously increased by 150 μM PA compared to the group without PA (Figure 8B). In summary, HK-2 cells exposed to 150 μM PA successfully mimicked the HF condition in DN *in vitro*. The RSV efficacy on HK-2 cells exposed to 150 μM PA was assessed by administering a series of RSV concentrations (0–80 μM). As depicted in Figure 8C, across the tested concentrations (1.25, 2.5, 5, 10 and 20 μM), RSV did not exert any significant effects on cell viability. However, a notable decline in cell viability was observed at RSV concentrations of 40 and 80 μM, compared to the group without RSV. Moreover, RSV at concentrations ranging from 1.25 to 20 μM did not ameliorate the reduction in cell viability under HF environments, whereas higher doses of RSV (40 and 80 μM) and 20 μM LW6 markedly enhanced cell viability compared with the PA group (Figure 8D).

**FIGURE 8:**
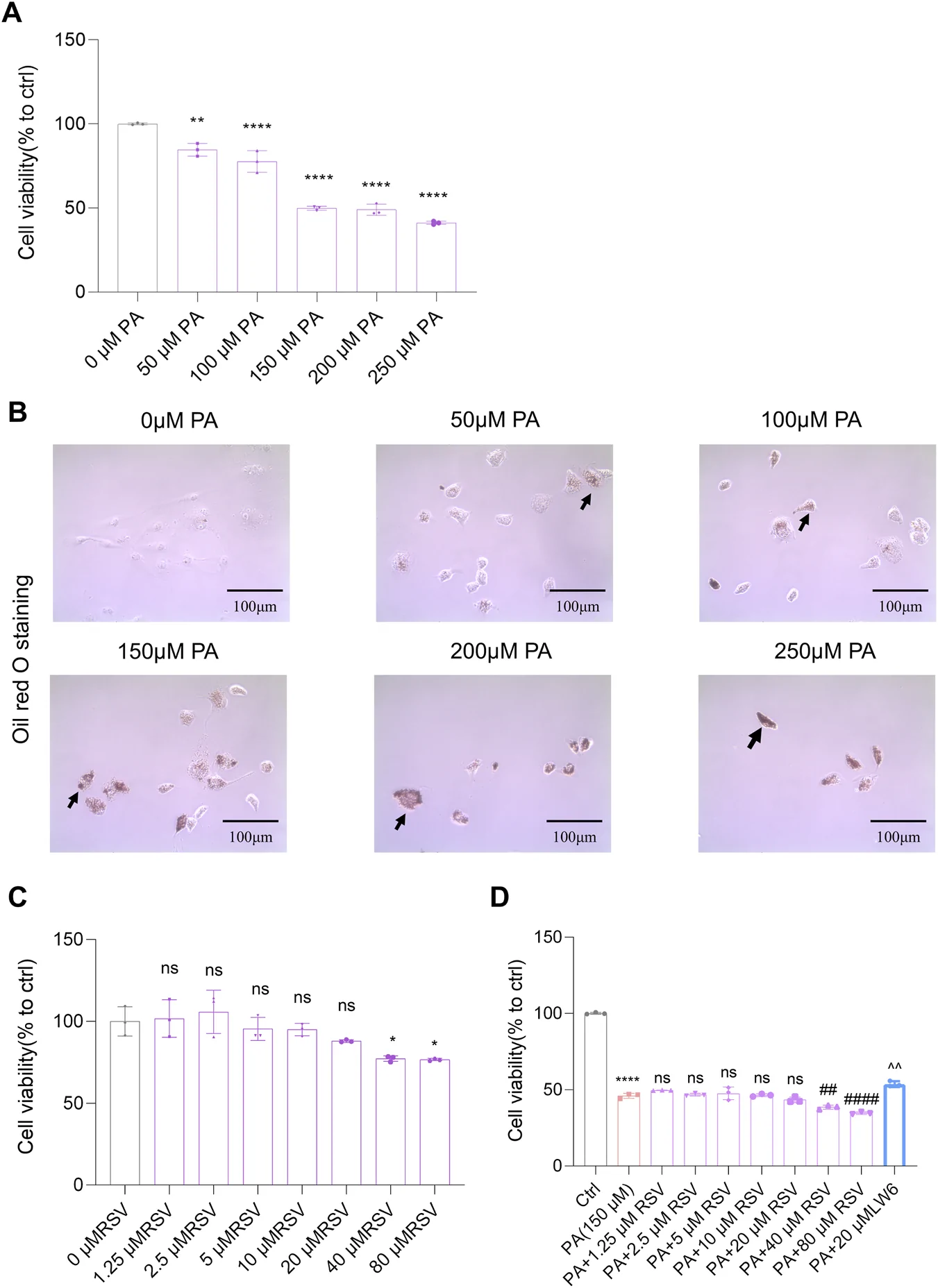
RSV alleviated PA-induced damage to HK-2 cells. **(A)** HK-2 cell viability across various PA concentrations (0, 50, 100, 150, 200 and 250 μM) assessed via CCK-8 assay. **(B)** Oil Red O staining. Black arrows indicate Oil Red O-positive lipid droplets. Magnification, ×200; Scale bar = 100 μm. **(C)** CCK-8 assay determined HK-2 cell viability with post-RSV treatment at different doses (0, 1.25, 2.5, 5, 10, 20, 40 and 80 μM). **(D)** Effects of LW6 and various concentrations of RSV on HK-2 cell viability in an HF condition by CCK-8 assay. ^*^ *P* < 0.05, ^**^ *P* < 0.01, ^***^ *P* < 0.001, ^****^ *P* < 0.0001 vs 0 μM PA, 0 μM RSV group and Ctrl group; ^#^ *P* < 0.05, ^##^ *P* < 0.01, ^###^ *P* < 0.001, ^####^ *P* < 0.0001 vs PA group; ^^^^ *P* < 0.01 vs PA group; ns: *P* > 0.05; n = 3.

#### 3.4.2 RSV alleviated lipid accumulation in PA-induced HK-2 cells through inhibiting HIF-1α

As noted above *in vivo* experiments, RSV suppressed renal HIF-1α expression in DN rats. To further validate whether RSV regulates glucolipid metabolism by HIF-1α suppression, intracellular lipid accumulation and HIF-1α expression were examined in HK-2 cells cultured in 150 μM PA and treated with either RSV (2.5–10 μM) or 20 μM LW6 (an HIF-1α inhibitor). As depicted in Figure 9A, PA treatment markedly enhanced the number of Oil Red O-positive lipid droplets compared with the control group. Fortunately, RSV reduced high-fat-induced lipid deposition dose-dependently. LW6 exhibited a similar effect comparable to that of 5 μM RSV, as reflected by the reduction in Oil Red O-positive area. Furthermore, protein expression and mRNA levels of HIF-1α in the PA group were markedly upregulated versus the control group, while RSV (2.5–10 μM) significantly reversed the increments (Figures 9B,C). LW6 only effectively decreased the HIF-1α protein expression, but failed to significantly decrease *HIF-1α* mRNA levels compared with the PA group (Figure 9C). Collectively, RSV attenuated lipid deposition in PA-induced HK-2 cells, which might be mediated by suppression of HIF-1α.

**FIGURE 9:**
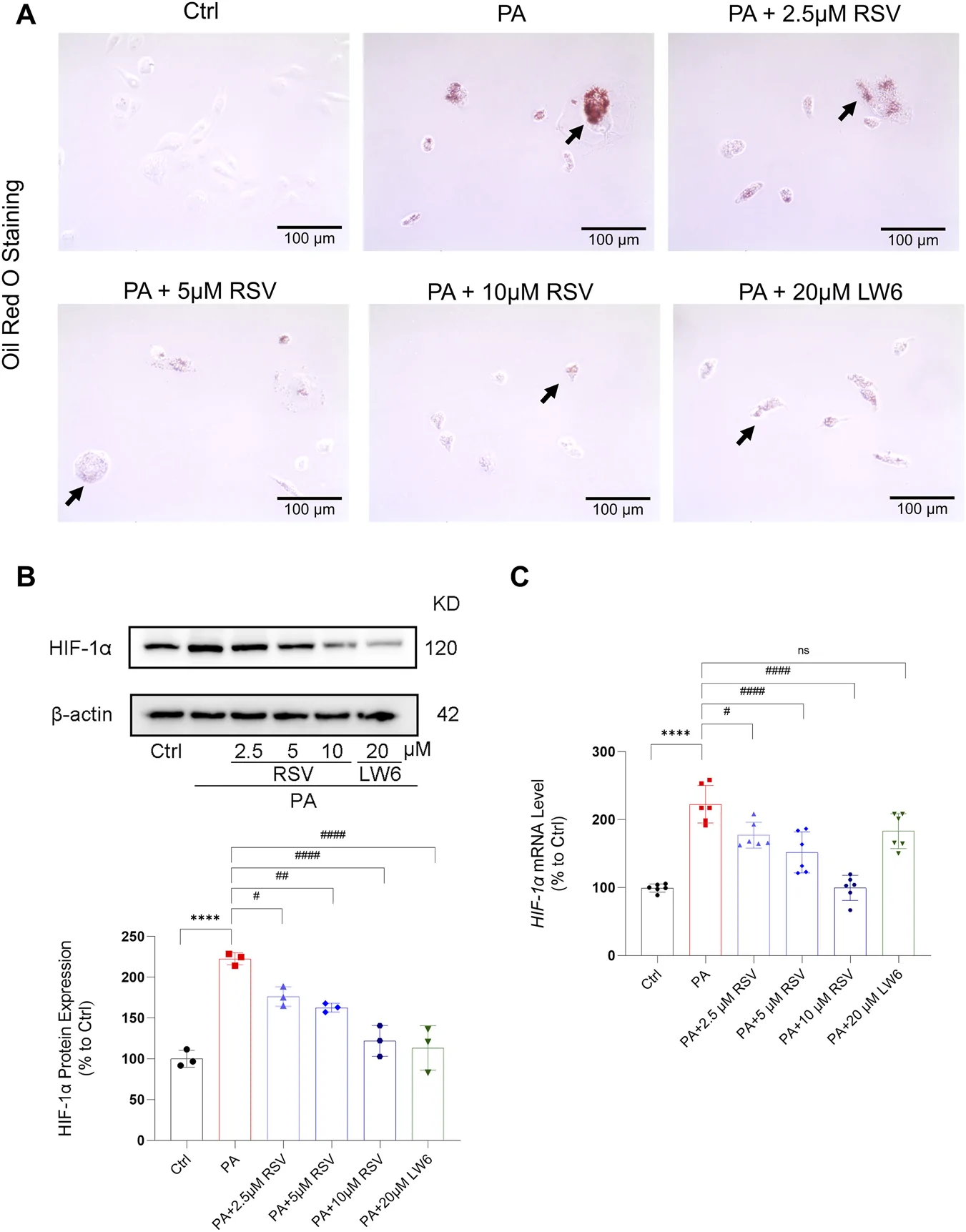
RSV alleviated lipid accumulation in PA-induced HK-2 cells through inhibiting HIF-1α. **(A)** Oil Red O staining. Black arrows indicate Oil Red O-positive lipid droplets. Magnification, ×200; Scale bar = 100 μm. **(B)** HIF-1α protein level in HK-2 cells was measured by Western blotting; n = 3. **(C)** *HIF-1*α mRNA level in HK-2 cells was measured by RT-qRCR. β-actin was used as the housekeeping gene; n = 6. ^*^ *P* < 0.05, ^**^ *P* < 0.01, ^***^ *P* < 0.001, ^****^ *P* < 0.0001 vs Ctrl group; ^#^ *P* < 0.05, ^##^ *P* < 0.01, ^###^ *P* < 0.001, ^####^ *P* < 0.0001 vs PA group; ns: *P* > 0.05.

#### 3.4.3 RSV regulated genes related to glucolipid metabolism in PA-induced HK-2 cells by suppressing HIF-1α

To further validate whether RSV exerts effects on glucolipid metabolism mediated by the modulation of downstream metabolic genes by suppressing HIF-1α, we evaluated protein expression and mRNA levels of SREBP1, SREBP2, LDLR, GLUT1 and INSIG2 in HK-2 cells. Firstly, mRNA levels of these five genes involved in glucolipid metabolism were examined. As depicted in Figures 10A–D, the mRNA levels of *SREBP1*, *SREBP2* and *GLUT1* were markedly elevated, while the *INSIG2* mRNA levels were decreased markedly in the PA group, relative to the control group. Luckily, LW6 and varying concentrations of RSV enhanced the *INSIG2* mRNA levels and reduced the mRNA levels of *SREBP1*, *SREBP2* and *GLUT1*, significantly, compared with the PA group. However, despite repeated attempts, we were unable to obtain reliable amplification signals for *LDLR* mRNA (Cq values ≥35). Accordingly, we proceeded to assess the protein expression of these five genes. As depicted in Figures 10E–J, protein expression of SREBP1, SREBP2, LDLR and GLUT1 in PA-pretreated HK-2 cells was markedly increased, while INSIG2 protein expression was markedly decreased in the PA group compared with the control group. However, the PA-induced dysregulation of these genes was significantly reversed by LW6 treatment. 2.5 μM RSV treatment failed to significantly decrease protein levels of GLUT1 and increase protein levels of INSIG2, whereas higher concentrations (5 and 10 μM) exerted significant and dose-dependent inhibitory effects on protein levels of GLUT1 and a stimulatory effect on protein levels of INSIG2. Meanwhile, various concentrations of RSV significantly downregulated protein levels of SREBP1, SREBP2, LDLR and GLUT1 compared with the PA groups. A correlation analysis between HIF-1α and INSIG2 protein levels (Supplementary Figure S3) revealed a significant negative correlation (r = −0.7944, *P* < 0.0001), which was inconsistent with previous reports describing a positive association (Kim et al., 2009). To further determine whether glucolipid metabolism disorders contribute to renal fibrosis, the protein levels of TGF-β1, a well-established pro-fibrotic marker, were assessed. As depicted in Figures 10E,K, the TGF-β1 protein expression was significantly upregulated in the PA group compared with the control group. Notably, the upregulation was markedly reversed by treatment with LW6 and RSV at all tested concentrations except 2.5 μM. These findings indicated that RSV regulated glucolipid metabolism, attenuating renal fibrosis in HK-2 cells by inhibiting HIF-1α and modulating downstream metabolic genes.

**FIGURE 10:**
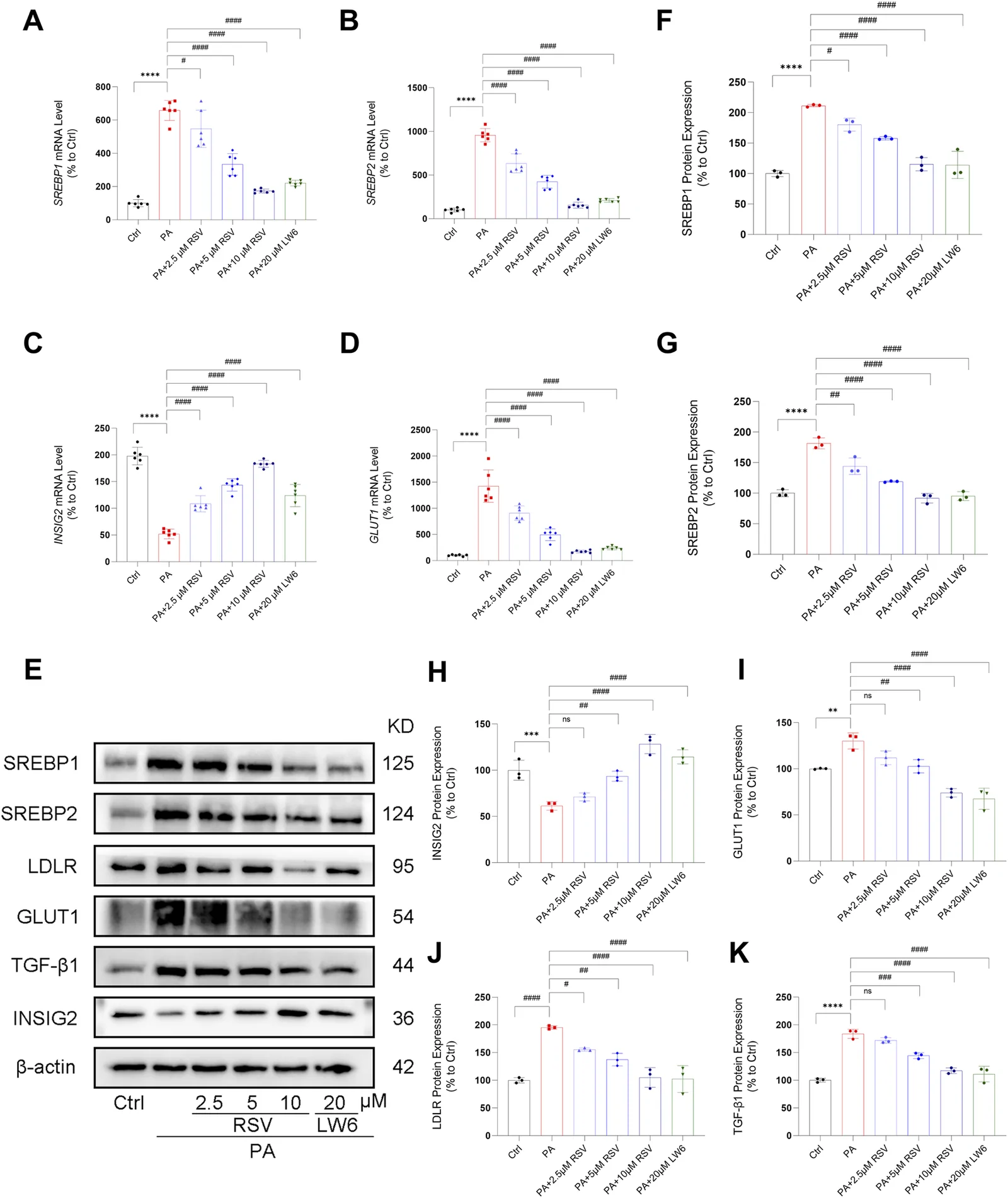
RSV regulated genes related to glucolipid metabolism in HK-2 cells by suppressing HIF-1α. **(A–D)** mRNA levels of *SREBP1*, *SREBP2*, *INSIG2* and *GLUT1*, respectively. β-actin was used as the housekeeping gene; n = 6. (E) Protein expression levels of SREBP1, SREBP2, LDLR, GLUT1, TGF-β1 and INSIG2 were determined via Western blot; n = 3. **(F–K)** Quantification of protein expression levels of SREBP1, SREBP2, INSIG2, GLUT1, LDLR and TGF-β1, respectively. ^*^ *P* < 0.05, ^**^ *P* < 0.01, ^***^ *P* < 0.001, ^****^ *P* < 0.0001 vs Ctrl group; ^#^ *P* < 0.05, ^##^ *P* < 0.01, ^###^ *P* < 0.001, ^####^ *P* < 0.0001 vs PA group; ns: *P* > 0.05.

## 4 Discussion

This study integrated network pharmacology, molecular docking and experimental validation, demonstrating that RSV ameliorated disturbances in renal glucolipid metabolism through reduction of AKT phosphorylation and suppression of the HIF-1α signaling in DN. We extended our previous *in vivo* study on the hepatic glucolipid metabolism of diabetic rats (Li et al., 2023) and offered novel insights into the mechanisms of RSV against DN in this study.

Network pharmacology identified AKT1, IL1B, INS, JUN, and STAT3 as the prioritized candidates of RSV in ameliorating glucolipid metabolism disorders in DN. Among them, AKT1 exhibited the highest binding affinity with RSV and is a central effector of glucolipid metabolism. The other four prioritized candidates (IL1B, STAT3, INS and JUN) were not pursued further because they are primarily associated with inflammatory signaling, insulin secretion, and transcriptional regulation, rather than directly with glucolipid metabolism (Veiras et al., 2022; Xuan et al., 2025; Welsh et al., 2010; Chen et al., 2025). These targets were not dismissed as irrelevant but warrant separate investigation in future studies. KEGG enrichment analysis revealed seven signaling pathways, among which the HIF-1 pathway displayed the greatest enrichment. Although HIF-1α was not a top-ranked candidate, it was selected because it is a critical downstream node of AKT governing hypoxia, inflammation and autophagy (Gonzalez et al., 2018), and AKT itself plays an important role in cell survival, proliferation, and insulin-dependent metabolic cell responses (Vasudevan and Garraway, 2010). We therefore postulated the AKT/HIF-1α pathway might serve as one of the key therapeutic targets of RSV in DN.

In the *in vivo* study, the renoprotective effects of RSV were tested in STZ-induced DN rats, consistent with our previous observations (Li et al., 2023; Feng et al., 2025). STZ combined with HFD induction model recapitulates metabolic features and natural progression observed in human T2DM, and generates stable and long-lasting hyperglycemia (Nath et al., 2017). Approximately 4 weeks after STZ injection, consistent with our findings in the DN group, renal injury characterized by increased albuminuria, elevated kidney index, and pathological alterations can be observed (Noshahr et al., 2020). Hyperglycemia is a primary driver of DN onset and progression, while SCr and BUN are critical biomarkers for renal function. A dose-response meta-analysis reported that RSV markedly improves the mesangial index, glomerular hypertrophy, SCr, BUN, 24-h urinary protein, blood glucose and KI (Liu et al., 2024). In this study, RSV did not exert marked influences on body weight and KI, but FBG, BUN and SCr were significantly reduced, alleviating renal injury and functional impairment. Moreover, glomerular lesions and renal glycogen accumulation were ameliorated after RSV treatment. Notably, RSV protected DN rats without noticeable systemic side effects. These findings suggest that RSV regulates glucolipid metabolism and attenuates pathological glomerular changes in DN.

To simulate the high-fat environment of DN *in vitro*, HK-2 cells received a range of PA concentrations (0–250 μM). PA at 150 μM induced a notable decline in cell viability while still maintaining sufficient cell viability for subsequent analysis. This result was consistent with a previous study using PA at 150 μM to induce lipotoxicity and oxidative injury in HK-2 cells (Ding et al., 2025). Furthermore, 150 μM PA markedly increased Oil Red O-positive lipid droplets, confirming successful induction of intracellular lipid deposition. This concentration provided sufficient lipotoxic stress for modeling metabolic disorders and adequate cellular viability for detecting RSV effects. RSV at 1.25–20 μM did not significantly affect cell viability under normal culture conditions, whereas 40 and 80 μM significantly reduced cell viability. Therefore, 2.5, 5 and 10 μM RSV were selected for subsequent investigations. However, RSV at various concentrations did not markedly increase cell viability under a high-fat environment, probably associated with PA-induced comprehensive stress, including lipid accumulation, inflammation, cell death and oxidative stress (Ceja-Galicia et al., 2025). In HK-2 cells exposed to H_2_O_2_, RSV treatment (5, 10 and 20 μM) also decreased cell viability significantly (Luo et al., 2025). Although our results showed that RSV suppressed HIF-1α expression and reduced lipid deposition, its protective effect might be insufficient to restore overall cell viability.

To further explore the mechanism by which RSV improves glucolipid metabolism in DN, we measured the effects of RSV on HIF-1α expression and compared them with those of LW6. Sustained hyperglycemia and hyperlipidemia stimulate oxygen consumption, leading to renal hypoxia. Hypoxia in renal tubules results in renal tubular atrophy and interstitial fibrosis, which accelerates glomerular lesions in DN and aggravates metabolic disorders. HIFs are essential mediators of adaptive responses to hypoxia. Under hypoxic conditions, the α-subunit undergoes stabilization and is primarily expressed in tubular cells to initiate an adaptive response to hypoxia (Qiyan et al., 2024). An SGLT2 (sodium-glucose cotransporter 2) inhibitor, Luseogliflozin, has been shown to decrease cortical tubular mitochondrial oxygen consumption and ameliorate renal damage by suppressing HIF-1α expression (Bessho et al., 2019). In addition, similar to our current results, HIF-1α knockout or inhibition mitigates mesangial matrix expansion, glomerulosclerosis, tubulointerstitial inflammation and fibrosis (Hu et al., 2017; Baumann et al., 2016). LW6 lowered HIF-1α protein levels through enhancing its degradation instead of interfering with its transcription (Lee et al., 2010), so *HIF-1α* mRNA levels in PA-treated HK-2 cells were not markedly decreased by LW6 in our study. However, the role of HIF-1α in DN remains controversial. Despite diabetes-induced renal hypoxia, accumulating evidence suggests that HIF-1α activation ameliorates renal mitochondrial dysfunction and improves renal function (Nordquist et al., 2015; Sugahara et al., 2020). HIF-1α supports cell survival via activating autophagy to moderate oxidative damage under mild hypoxia conditions, while severe and chronic hypoxia triggers cell death and inflammation through exacerbating abnormal autophagy and glycolysis (Bi et al., 2024). Therefore, whether HIF-1α serves as a positive or detrimental factor depends on the severity and duration of hypoxia.

In this study, we also found that RSV upregulated *Ldlr* mRNA levels *in vivo* and INSIG2 levels *in vitro*, and downregulated SREBP1, SREBP2, LDLR, GLUT1 and TGF-β1 levels *in vitro* via inhibiting HIF-1α. When intracellular cholesterol is high, INSIG2 accumulation inactivates SREBPs, preventing the excretion of SCAP-SREBP complexes from the endoplasmic reticulum, which downregulates LDLR protein via reduced SREBP2-mediated transactivation of the LDLR gene (Kim et al., 2009; Bjune et al., 2019). Whereas the previous study suggests a positive regulation between HIF-1α and INSIG2 (Kim et al., 2009), our correlation analysis revealed a significant negative correlation between HIF-1α and INSIG2 protein levels. This discrepancy warrants further investigation. Notably, RSV treatment markedly increased renal *Ldlr* mRNA levels in DN rats, whereas in PA-treated HK-2 cells, RSV downregulated LDLR protein expression and *LDLR* mRNA was not reliably detectable (Cq values ≥ 35). This *in vivo* and *in vitro* discordance may be explained by post-transcriptional regulation of LDLR by PCSK9 and fundamental differences in the regulatory mechanisms governing LDLR expression between the two experimental systems. LDLR protein levels are subject to post-transcriptional regulation by PCSK9, which binds the epidermal growth factor A domain of LDLR and targets the receptor for lysosomal degradation without affecting LDLR mRNA levels (Sundararaman et al., 2021). HK-2 cells exposed to PCSK9-containing medium showed reduced LDLR protein levels without significant changes in *LDLR* mRNA (Byun et al., 2022). While RSV downregulates PCSK9 expression in hepatocytes (Adorni et al., 2020), evidence in the kidney remains limited. If a similar effect occurs in the kidney, RSV could increase LDLR protein by reducing its degradation. The net effect on LDLR protein therefore depends on the balance between transcriptional activation and post-transcriptional degradation. In addition, tissue-specific and cell type-specific regulatory mechanisms may also contribute to the divergent LDLR expression. Previous reports have indicated that RSV-mediated LDLR upregulation serves as a protective adaptive response to metabolic stress, promoting cholesterol clearance and reducing intracellular lipid accumulation (Yashiro et al., 2012; Jing et al., 2019). Conversely, RSV directly downregulated LDLR in HK-2 cells, likely as a direct protective mechanism to limit cholesterol uptake under lipotoxic stress (Zhang et al., 2015). Rather than representing contradictory findings, these complementary observations highlight the multi-layered regulatory mechanisms of RSV-mediated renoprotection. For lipid synthesis, the SREBP family plays an important role. SREBP-1c and SREBP-2 govern fatty acid and cholesterol synthesis, respectively. Aberrant tubule cholesterol metabolism might induce cholesterol crystal formation, thereby promoting renal inflammation and fibrosis (Yang et al., 2025). SREBP1 activation facilitates epithelial-mesenchymal transition through upregulation of TGF-β1, which was consistent with our results (Gabitova-Cornell et al., 2020). For glucose handling, GLUTs regulate glucose metabolism and inflammatory responses, with high sensitivity to intracellular signals and metabolites (Wang et al., 2024). Hypoxia induces the transcription of *Glut1*, but we found that RSV reduced the increased GLUT1 levels both *in vivo* and *in vitro*. GLUT1 suppression also interferes with TGF-β1-induced fibrogenesis, comprising cell proliferation and the production of profibrotic mediators (Andrianifahanana et al., 2016). Therefore, RSV reduced GLUT1 levels, potentially affecting anaerobic metabolism. Furthermore, RSV increased INSIG-2 expression and *Ldlr* mRNA levels, while decreasing SREBP1, SREBP2 and LDLR levels to modulate cholesterol synthesis and uptake. Finally, RSV inhibited TGF-β1 expression, leading to a reduction of renal fibrosis.

Overall, we extend our previously established RSV-mediated AKT/HIF-1α mechanism from liver to the kidney in DN, and further identify this axis as a key checkpoint through network pharmacology-guided target discovery, which was validated in both *in vivo* and *in vitro* experiments. However, there are several limitations. First, only male rats were used, and n = 3 per group in the *in vivo* mechanistic analyses (Figure 7) was due to limited cortical tissue availability and quality-control exclusion. Although cortical tissue was used to minimize cortico-medullary heterogeneity, HIF-1α expression still varies between tubular and glomerular compartments (Sharma et al., 2020). Second, total AKT was not measured, so reduced p-AKT may reflect either decreased phosphorylation or reduced total AKT abundance. Moreover, whether AKT dephosphorylation is required for RSV-mediated HIF-1α suppression was not tested using AKT inhibitors or AKT siRNA, and further blots with p-AKT/total-AKT ratios are needed. Third, although the *in vitro* experiments with LW6 support a role for HIF-1α, the *in vivo* causal relationship between HIF-1α suppression and renoprotection was not tested by loss-of-function approaches. Fourth, the upstream-downstream relationship between HIF-1α and INSIG2 remains associative and requires immunoprecipitation and other similar methods. Lastly, effective *in vitro* RSV concentrations (2.5–10 μM) exceed those achievable *in vivo* after oral administration, and renal tissue RSV concentrations after oral dosing are limited (Abd El-Mohsen et al., 2006; Sergides et al., 2016). Low oral bioavailability may be attributed to the extensive intestinal and hepatic metabolisms (Walle et al., 2004).

To advance RSV toward clinical use in DN, several steps will be required. First, the causal role of HIF-1α in mediating RSV’s renoprotection should be validated using *Hif-1α* siRNA or CRISPR-mediated knockout. Second, the therapeutic potential of RSV as an adjunct to current DN therapies, such as SGLT2 inhibitors, GLP-1R (glucagon-like peptide-1 receptor) agonists and lipid-lowering agents, warrants investigation. Third, given RSV’s well-established efficacy and safety but low oral bioavailability, kidney-targeted nanodelivery systems, such as pH-responsive liposomes (Yang et al., 2026), or prodrug strategies should be developed to enhance renal exposure and therapeutic efficacy.

## 5 Conclusion

Collectively, as depicted in Figure 1, RSV alleviates glucolipid metabolism disorders in DN. This effect is associated with suppression of the HIF-1α pathway and its downstream targets related to glucolipid metabolism, in both STZ+HFD-induced diabetic rats and PA-induced HK-2 cells, thereby potentially ameliorating anaerobic metabolism, cholesterol processing and renal fibrosis. However, the causal contribution of HIF-1α suppression to these *in vivo* effects remains to be confirmed. These findings offer a mechanistic foundation for the therapeutic application of RSV in DN and highlight the value of integrating computational prediction with experimental validation in drug discovery research.

## Data Availability

The datasets presented in this study can be found in online repositories. The names of the repository/repositories and accession number(s) can be found in the article/Supplementary Material.
