# Supplementary material for: Resveratrol alleviates glucolipid metabolism disorders in diabetic nephropathy via the HIF-1α pathway: a network pharmacology and experimental validation study
Source: Front Pharmacol. 2026 Oct 30;17:1971426. doi: 10.3389/fphar.2026.1971426 (PMC13759819; doi:10.3389/fphar.2026.1971426)
Supplement: Supplementary file 1 [file DataSheet1.pdf]

Supplementary Figures

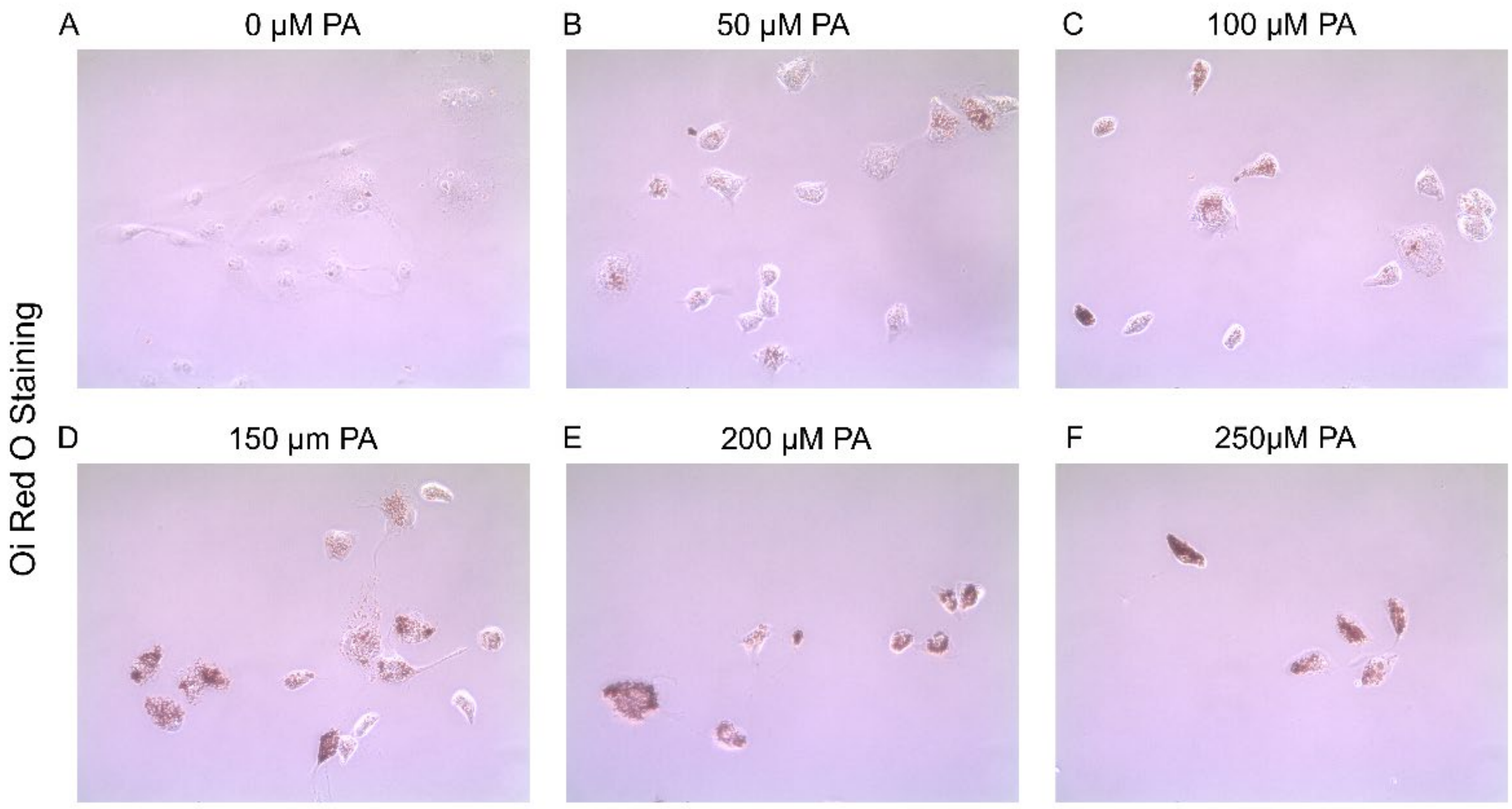

Supplementary Figure S1 Representative Oil Red O staining images of HK-2 cells treated with different concentrations of PA (PA: 0, 50, 100, 150, 200, and 250 μM). (A–F) Corresponding to each concentration. Magnification, ×200; Scale bar = 100 μm.

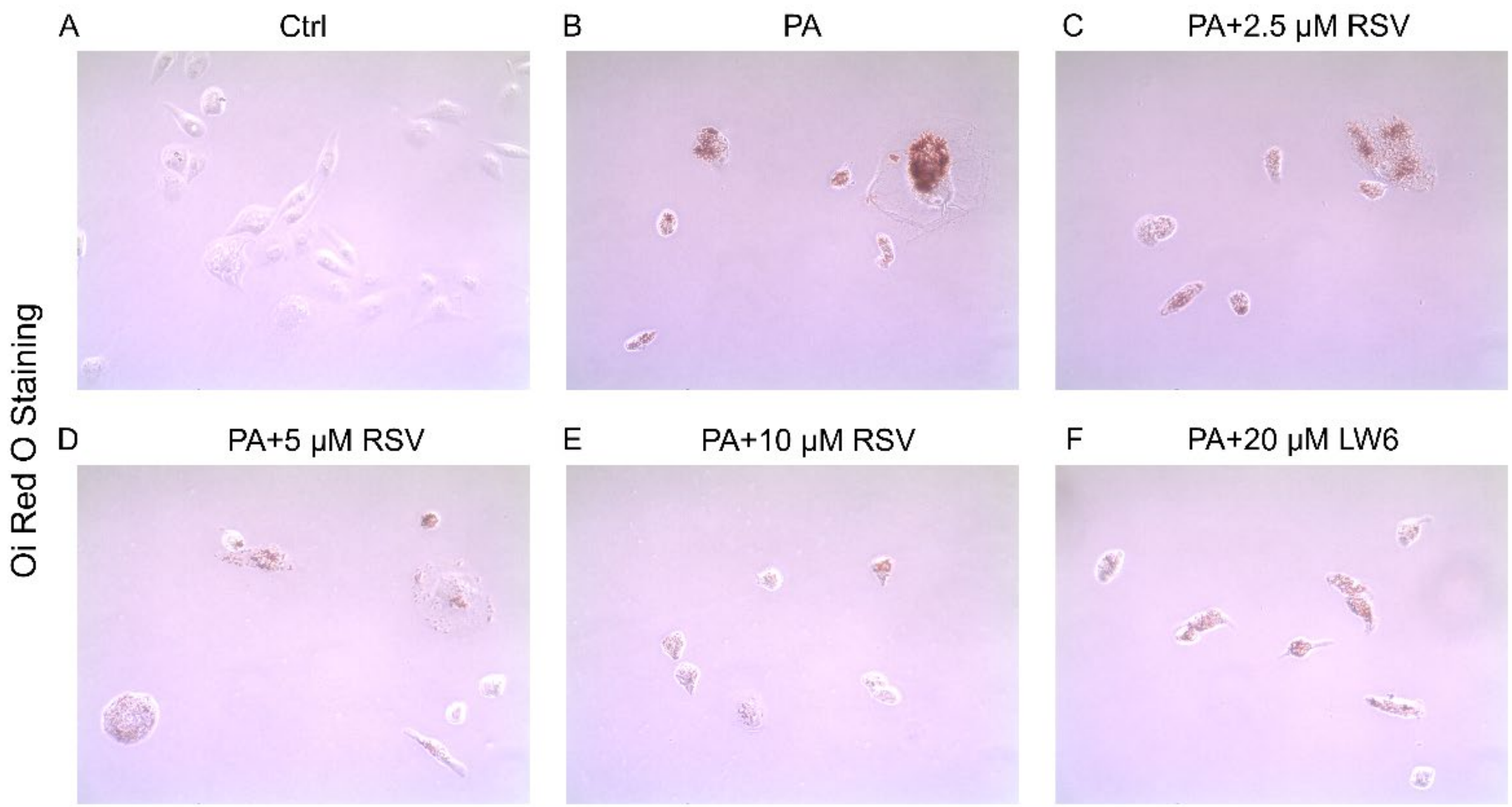

Supplementary Figure S2 Representative Oil Red O staining images of HK-2 cells under different treatments. (A) Ctrl; (B) PA (150 μM); (C) PA + 2.5 μM RSV; (D) PA + 5 μM RSV; (E) PA + 10 μM RSV; (F) PA + 20 μM LW6. Magnification, ×200; Scale bar = 100 μm.

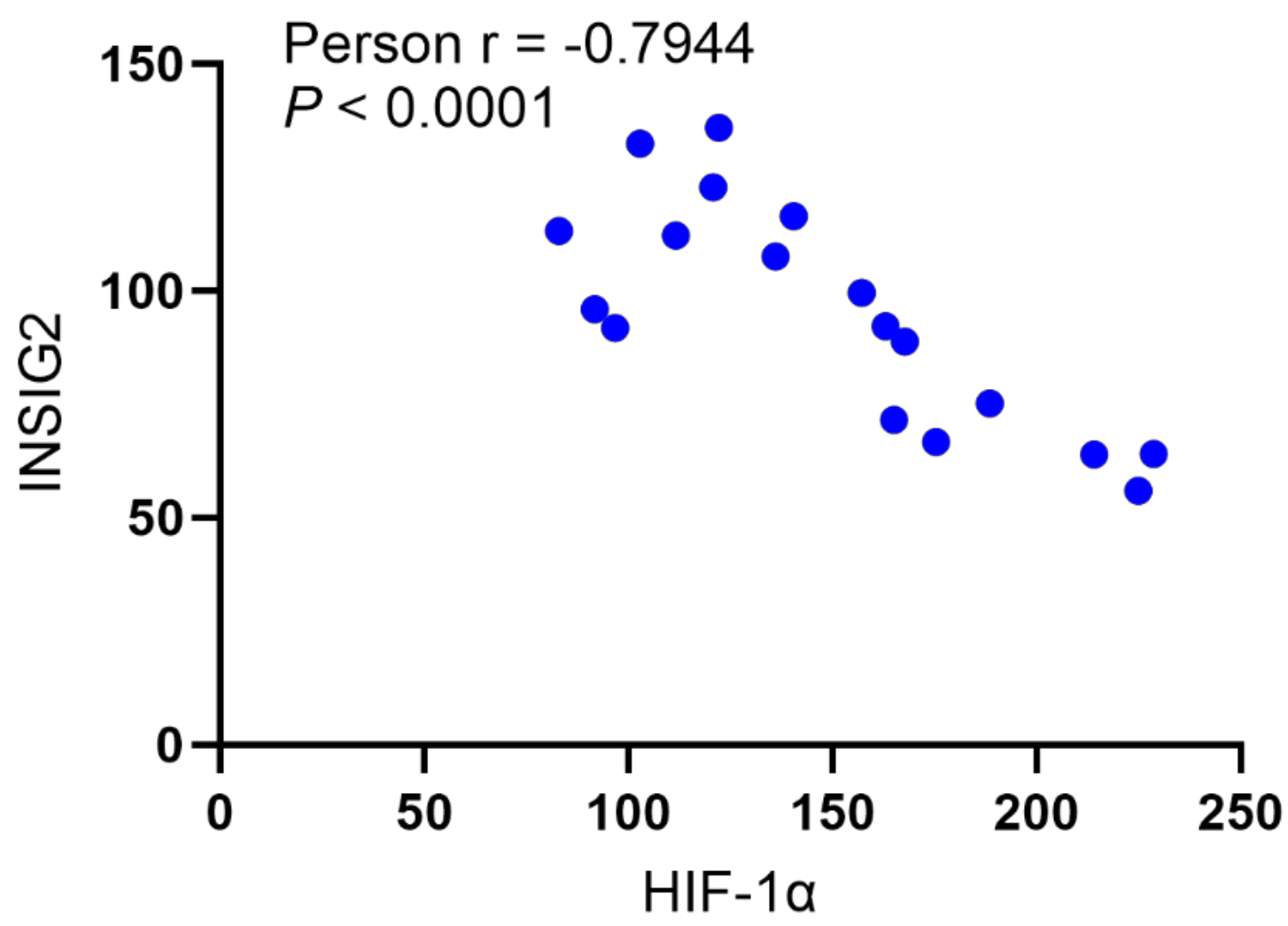

Supplementary Figure S3 Correlation between HIF-1α and INSIG2 protein levels.
