# Supplementary material for: Resveratrol alleviates glucolipid metabolism disorders in diabetic nephropathy via the HIF-1α pathway: a network pharmacology and experimental validation study
Source: Front Pharmacol. 2026 Oct 30;17:1971426. doi: 10.3389/fphar.2026.1971426 (PMC13759819; doi:10.3389/fphar.2026.1971426)
Supplement: Supplementary file 2 [file DataSheet2.pdf]

# Supplementary Table

Supplementary Table S1. Exact *P* values and effect sizes for all pairwise comparisons.

| Figure | Comparison | Metric | *P* value | Hedges' \|g\| |
|---|---|---|---|---|
| Fig. 5A | NC vs DM | Body weight | 0.0149* | 1.3532 |
| | DM vs DR | Body weight | 0.9289 | 0.0523 |
| Fig. 5B | NC vs DM | Kidney index | 0.0006*** | 2.1038 |
| | DM vs DR | Kidney index | 0.6500 | 0.2036 |
| Fig. 5C | NC vs DM | FBG | <0.0001**** | 8.3669 |
| | DM vs DR | FBG | 0.0009### | 1.8193 |
| Fig. 5D | NC vs DM | BUN | 0.0002*** | 2.1951 |
| | DM vs DR | BUN | 0.0106# | 1.2948 |
| Fig. 5E | NC vs DM | SCr | 0.0232* | 1.3951 |
| | DM vs DR | SCr | 0.0019## | 1.9079 |
| Fig. 7B | NC vs DM | HIF-1α protein (IF) | <0.0001**** | 7.3399 |
| | DM vs DR | HIF-1α protein (IF) | <0.0001#### | 6.6610 |
| Fig. 7C | NC vs DM | HIF-1α protein (WB) | 0.0159* | 5.7490 |
| | DM vs DR | HIF-1α protein (WB) | 0.0442# | 1.4202 |
| Fig. 7D | NC vs DM | p-AKT protein | 0.0166* | 2.0782 |
| | DM vs DR | p-AKT protein | 0.0443# | 2.4794 |
| Fig. 7E | NC vs DM | *Glut1* mRNA | <0.0001**** | 9.1606 |
| | DM vs DR | *Glut1* mRNA | 0.0003### | 4.6620 |
| Fig. 7F | NC vs DM | *Ldlr* mRNA | <0.0001**** | 10.8186 |
| | DM vs DR | *Ldlr* mRNA | <0.0001#### | 6.7212 |
| Fig. 8A | 0 μM PA vs 50 μM PA | Cell viability | 0.0014** | 4.6000 |
| | 0 μM PA vs 100 μM PA | Cell viability | <0.0001**** | 3.8896 |
| | 0 μM PA vs 150 μM PA | Cell viability | <0.0001**** | 43.4960 |
| | 0 μM PA vs 200 μM PA | Cell viability | <0.0001**** | 17.2560 |

| | | | | |
|---|---|---|---|---|
| | 0 μM PA vs 250 μM PA | Cell viability | <0.0001$^{****}$ | 59.0720 |
| Fig. 8C | 0 μM RSV vs 1.25 μM RSV | Cell viability | >0.9999 | 0.1345 |
| | 0 μM RSV vs 2.5 μM RSV | Cell viability | 0.9786 | 0.4045 |
| | 0 μM RSV vs 5 μM RSV | Cell viability | 0.9936 | 0.4563 |
| | 0 μM RSV vs 10 μM RSV | Cell viability | 0.9898 | 0.5806 |
| | 0 μM RSV vs 20 μM RSV | Cell viability | 0.5418 | 1.5053 |
| | 0 μM RSV vs 40 μM RSV | Cell viability | 0.0322$^{*}$ | 2.8212 |
| | 0 μM RSV vs 80 μM RSV | Cell viability | 0.0274$^{*}$ | 2.9266 |
| Fig. 8D | Ctrl vs PA | Cell viability | <0.0001$^{****}$ | 35.1858 |
| | PA vs PA + 1.25 μM RSV | Cell viability | 0.3273 | 2.4711 |
| | PA vs PA + 2.5 μM RSV | Cell viability | >0.9999 | 0.4626 |
| | PA vs PA + 5 μM RSV | Cell viability | 0.9795 | 0.3986 |
| | PA vs PA + 10 μM RSV | Cell viability | >0.9999 | 0.2646 |
| | PA vs PA + 20 μM RSV | Cell viability | 0.7174 | 1.2292 |
| | PA vs PA + 40 μM RSV | Cell viability | 0.0016$^{\#\#}$ | 3.9705 |
| | PA vs PA + 80 μM RSV | Cell viability | <0.0001$^{\#\#\#\#}$ | 7.1393 |
| | PA vs PA + LW6 | Cell viability | 0.0010$^{\wedge\wedge}$ | 3.5274 |
| Fig. 9B | Ctrl vs PA | HIF-1α protein | <0.0001$^{****}$ | 10.8420 |
| | PA vs PA + 2.5 μM RSV | HIF-1α protein | 0.0299$^{\#}$ | 3.7405 |
| | PA vs PA + 5 μM RSV | HIF-1α protein | 0.0048$^{\#\#}$ | 7.3427 |
| | PA vs PA + 10 μM RSV | HIF-1α protein | <0.0001$^{\#\#\#\#}$ | 5.6044 |
| | PA vs PA + LW6 | HIF-1α protein | <0.0001$^{\#\#\#\#}$ | 4.3616 |
| Fig. 9C | Ctrl vs PA | *HIF-1α* mRNA | <0.0001$^{****}$ | 5.7156 |
| | PA vs PA + 2.5 μM RSV | *HIF-1α* mRNA | 0.0171$^{\#}$ | 1.7765 |
| | PA vs PA + 5 μM RSV | *HIF-1α* mRNA | <0.0001$^{\#\#\#\#}$ | 2.2731 |
| | PA vs PA + 10 μM RSV | *HIF-1α* mRNA | <0.0001$^{\#\#\#\#}$ | 4.8467 |
| | PA vs PA + LW6 | *HIF-1α* mRNA | 0.0510 | 1.3701 |
| Fig. 10A | Ctrl vs PA | *SREBP1* mRNA | <0.0001$^{****}$ | 11.3749 |
| | PA vs PA+2.5 μM RSV | *SREBP1* mRNA | 0.0342$^{\#}$ | 1.1306 |

| | | | | |
|---|---|---|---|---|
| | PA vs PA+5 μM RSV | *SREBP1* mRNA | <0.0001#### | 4.7848 |
| | PA vs PA+10 μM RSV | *SREBP1* mRNA | <0.0001#### | 10.3086 |
| | PA vs PA+20 μM LW6 | *SREBP1* mRNA | <0.0001#### | 9.1184 |
| Fig. 10B | Ctrl vs PA | *SREBP2* mRNA | <0.0001**** | 14.1956 |
| | PA vs PA+2.5 μM RSV | *SREBP2* mRNA | <0.0001#### | 3.1656 |
| | PA vs PA+5 μM RSV | *SREBP2* mRNA | <0.0001#### | 6.6187 |
| | PA vs PA+10 μM RSV | *SREBP2* mRNA | <0.0001#### | 12.5931 |
| | PA vs PA+20 μM LW6 | *SREBP2* mRNA | <0.0001#### | 12.4144 |
| Fig. 10C | Ctrl vs PA | *INSIG2* mRNA | <0.0001**** | 10.1277 |
| | PA vs PA+2.5 μM RSV | *INSIG2* mRNA | <0.0001#### | 4.1812 |
| | PA vs PA+5 μM RSV | *INSIG2* mRNA | <0.0001#### | 8.1573 |
| | PA vs PA+10 μM RSV | *INSIG2* mRNA | <0.0001#### | 15.3401 |
| | PA vs PA+20 μM LW6 | *INSIG2* mRNA | <0.0001#### | 4.1153 |
| Fig. 10D | Ctrl vs PA | *GLUT1* mRNA | <0.0001**** | 5.5546 |
| | PA vs PA+2.5 μM RSV | *GLUT1* mRNA | <0.0001#### | 1.9740 |
| | PA vs PA+5 μM RSV | *GLUT1* mRNA | <0.0001#### | 3.6683 |
| | PA vs PA+10 μM RSV | *GLUT1* mRNA | <0.0001#### | 5.2416 |
| | PA vs PA+20 μM LW6 | *GLUT1* mRNA | <0.0001#### | 4.9559 |
| Fig. 10F | Ctrl vs PA | SREBP1 protein | <0.0001**** | 22.5999 |
| | PA vs PA+2.5 μM RSV | SREBP1 protein | 0.0483* | 3.2547 |
| | PA vs PA+5 μM RSV | SREBP1 protein | <0.0001#### | 18.0685 |
| | PA vs PA+10 μM RSV | SREBP1 protein | <0.0001#### | 9.8470 |
| | PA vs PA+20 μM LW6 | SREBP1 protein | <0.0001#### | 4.9370 |
| Fig. 10G | Ctrl vs PA | SREBP2 protein | <0.0001**** | 8.7464 |
| | PA vs PA+2.5 μM RSV | SREBP2 protein | 0.0013## | 2.6360 |
| | PA vs PA+5 μM RSV | SREBP2 protein | <0.0001#### | 7.9176 |
| | PA vs PA+10 μM RSV | SREBP2 protein | <0.0001#### | 8.5848 |
| | PA vs PA+20 μM LW6 | SREBP2 protein | <0.0001#### | 8.5440 |
| Fig. 10H | Ctrl vs PA | INSIG2 protein | 0.0005*** | 4.7219 |

| | | | | |
|---|---|---|---|---|
| | PA vs PA+2.5 μM RSV | INSIG2 protein | 0.6322 | 4.2187 |
| | PA vs PA+5 μM RSV | INSIG2 protein | 0.0026$^{\#\#}$ | 7.8689 |
| | PA vs PA+10 μM RSV | INSIG2 protein | <0.0001$^{\#\#\#\#}$ | 7.9730 |
| | PA vs PA+20 μM LW6 | INSIG2 protein | <0.0001$^{\#\#\#\#}$ | 8.7064 |
| Fig. 10I | Ctrl vs PA | GLUT1 protein | 0.0036$^{**}$ | 3.9344 |
| | PA vs PA+2.5 μM RSV | GLUT1 protein | 0.0923 | 1.8064 |
| | PA vs PA+5 μM RSV | GLUT1 protein | 0.0076$^{\#\#}$ | 2.7592 |
| | PA vs PA+10 μM RSV | GLUT1 protein | <0.0001$^{\#\#\#\#}$ | 6.5112 |
| | PA vs PA+20 μM LW6 | GLUT1 protein | <0.0001$^{\#\#\#\#}$ | 4.9272 |
| Fig. 10J | Ctrl vs PA | LDLR protein | <0.0001$^{****}$ | 18.8064 |
| | PA vs PA+2.5 μM RSV | LDLR protein | 0.0310$^{\#}$ | 10.6824 |
| | PA vs PA+5 μM RSV | LDLR protein | 0.0020$^{\#\#}$ | 5.5736 |
| | PA vs PA+10 μM RSV | LDLR protein | <0.0001$^{\#\#\#\#}$ | 5.6720 |
| | PA vs PA+20 μM LW6 | LDLR protein | <0.0001$^{\#\#\#\#}$ | 4.2992 |
| Fig. 10K | Ctrl vs PA | TGF-β1 protein | <0.0001$^{****}$ | 10.6309 |
| | PA vs PA+2.5 μM RSV | TGF-β1 protein | 0.4436 | 1.3980 |
| | PA vs PA+5 μM RSV | TGF-β1 protein | 0.0005$^{\#\#\#}$ | 4.5672 |
| | PA vs PA+10 μM RSV | TGF-β1 protein | <0.0001$^{\#\#\#\#}$ | 7.7640 |
| | PA vs PA+20 μM LW6 | TGF-β1 protein | <0.0001$^{\#\#\#\#}$ | 5.0790 |

Data were analyzed using one-way ANOVA followed by LSD post hoc test. Exact P-values are reported. Hedges' |g| are provided for pairwise comparisons; Hedges' |g| was corrected for small sample size (n = 3, J = 0.8000; n = 6, J = 0.9231). $^{*}P < 0.05$, $^{**}P < 0.01$, $^{***}P < 0.001$, $^{****}P < 0.0001$ vs NC / Ctrl group; $^{\#}P < 0.05$, $^{\#\#}P < 0.01$, $^{\#\#\#}P < 0.001$, $^{\#\#\#\#}P < 0.0001$ vs DM / PA group; $^{\wedge\wedge}P < 0.01$vs PA group.
